# Chromosome-level reference genome assembly provides insights into the evolution of *Pennisetum alopecuroides*

**DOI:** 10.3389/fpls.2023.1195479

**Published:** 2023-08-23

**Authors:** Ke Teng, Qiang Guo, Lingyun Liu, Yidi Guo, Yue Xu, Xincun Hou, Wenjun Teng, Hui Zhang, Chunqiao Zhao, Yuesen Yue, Haifeng Wen, Juying Wu, Xifeng Fan

**Affiliations:** Institute of Grassland, Flowers, and Ecology, Beijing Academy of Agriculture and Forestry Sciences, Beijing, China

**Keywords:** *P. alopecuroides*, *de novo* assembly, comparative genomics, whole-genome duplication, forage grass

## Abstract

*Pennisetum alopecuroides* is an important forage grass resource, which plays a vital role in ecological environment improvement. Therefore, the acquisition of *P. alopecuroides* genome resources is conducive to the study of the adaptability of *Pennisetum* species in ecological remediation and forage breeding development. Here we assembled a *P. alopecuroides* cv. 'Liqiu' genome at the chromosome level with a size of approximately 845.71 Mb, contig N50 of 84.83Mb, and genome integrity of 99.13% as assessed by CEGMA. A total of 833.41-Mb sequences were mounted on nine chromosomes by Hi-C technology. In total, 60.66% of the repetitive sequences and 34,312 genes were predicted. The genomic evolution analysis showed that *P. alopecuroides* cv. 'Liqiu' was isolated from Setaria 7.53–13.80 million years ago and from Cenchrus 5.33–8.99 million years ago, respectively. The whole-genome event analysis showed that *P. alopecuroides* cv. 'Liqiu' underwent two whole-genome duplication (WGD) events in the evolution process, and the duplication events occurred at a similar time to that of *Oryza sativa* and *Setaria viridis*. The completion of the genome sequencing of *P. alopecuroides* cv. 'Liqiu' provides data support for mining high-quality genetic resources of *P. alopecuroides* and provides a theoretical basis for the origin and evolutionary characteristics of *Pennisetum*.

## 1 Introduction

Pennisetum, as an excellent forage, has been widely used all over the world (Hou et al., 2022). The main cultivated species in China are perennial elephant grass (*P. purpureum*), pearl millet [*Pennisetum glaucum* (L.) R. Br.], and hybrid *Pennisetum* (*P. glaucum* × *P. purpureum*). Pennisetum plants have strong stress resistance, moderate salt and alkaline resistance, low water resource consumption, and landscape value (Yue et al., 2020). It could also be used in soil and water conservation, sand fixation, and landscape construction, which means that it has a good ecological restoration and application prospect.

With the rapid improvement of sequencing technology, whole-genome sequencing information have been obtained from more and more species (Marks et al., 2021). At present, whole-genome sequencing has been completed in lots of plants (Zheng et al., 2021). Traditional horticultural plant genomes, such as *Helianthus annuus* (Badouin et al., 2017), *Magnolia biondii* (Dong et al., 2021), *Prunus yedoensis* (Baek et al., 2018), *Phalaenopsis* (Cai et al., 2015), *Dendrobium offificinale* (Liang et al., 2015), and *Chrysanthemum nankingense* (Song et al., 2018), have been obtained with high quality. The completion of horticultural plant genome sequencing lays a foundation for the analysis of plant type, flowering, growth and development, and other regulatory mechanisms so as to achieve accurate breeding. In recent years, genome assembly and annotation in Gramineae have been enriched (International Brachypodium, I, 2010; Byrne et al., 2015; Lovell et al., 2018; Huang et al., 2019; Mitros et al., 2020; Wang et al., 2021). The quality of chromosome assembly has also been improved in species closely related to Pennisetum. *Setaria italica* has completed chromosome assembly, which contig N50 increased to 11.2 Mb compared with the prior 1.6 Mb (Bennetzen et al., 2012; Mamidi et al., 2020). The elephant grass (*Cenchrus purpureus*) (2*n* = 4*x* = 28, AABB) was sequenced with contig N50 of 2.65 Mb higher than the first published genome of *C. purpureus* cv. Purple (contig N50 of 1.83 Mb) (Yan et al., 2020; Zhang et al., 2022a). In addition, according to the whole-genome sequence of *Pennisetum glaucum* (L.) R. Br., syn. *Cenchrus americanus* (L.) Morrone (2*n* = 2*x* = 14, AA), NAC transcription factors were found to play an important role in drought tolerance (Dudhate et al., 2021; Yan et al., 2023). Recently, a predominantly Pennisetum multiple omics database platform (Milletdb, http://milletdb.novogene.com/) was established (Sun et al., 2023). However, the genome still needs to increase the number of grass plants to further understand the evolutionary history and find more resistance candidate genes to improve breeding.

*Pennisetum* is a kind of forage (Tenorio and Roque, 2015), food crop (Xu et al., 2021), energy grass (Hayat, 2020), and ecological grass (Gallego et al., 2015). *Pennisetum* is broadly used as an ecological grass around the world (Zhang et al., 2016), such as *Pennisetum alopecuroides* cv. Rubrum (Liu et al., 2022) and *Pennisetum alopecuroides* cv. 'Little Bunny' (Guo et al., 2022). In this study, *P. alopecuroides* cv. 'Liqiu' was selected for genome assembly, which has several advantages, including longer green periods, outstanding cold resistance, and better palatability. It could be used for ecological restoration or landscape construction of barren mountains and slopes, and it could also be used for landscaping.

Here we used PacBio, Illumina, and Hi-C auxiliary assembly strategies to assemble a high-quality genome. The reference genome could provide the basis for functional genomics and improve the molecular basis. In addition, candidate functional genes could be identified to reveal the adaptation mechanism of *Pennisetum* to different adversities.

## 2 Materials and methods

### 2.1 Sampling collection and sequencing

**Figure 1:**
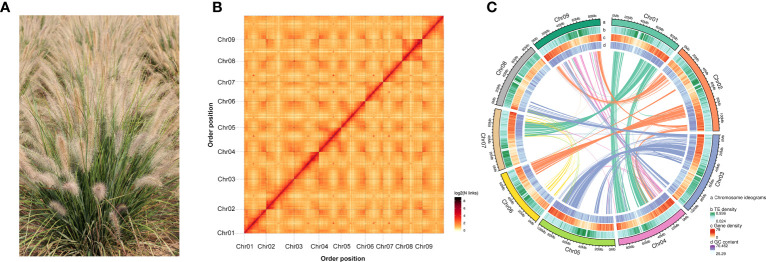
Genome assembly and situation of chromosomal collinearity. **(A)** Photograph of *P. alopecuroides* morphology. **(B)** A heat map of chromosome interactions in the *P. alopecuroides* genome shows the interactions between nine chromosomes (Chr1–9). Both horizontal and vertical coordinates represent the order of each bin on the corresponding chromosome group. **(C)** *P. alopecuroides* genome circle map. The outermost layer shows the number of chromosomes, from outside to inside, gene density, repeat sequence density, GC content, and collinearity.

*P. alopecuroides* cv. 'Liqiu' (**Figure 1A**) was identified and cultivated at the Institute of Grassland, Flowers, and Ecology, Beijing Academy of Agriculture and Forestry Sciences (Beijing, China). Healthy leaves were collected from the plants of varieties growing in National Precision Agriculture Research Demonstration Base, Changping District, Beijing, China (116°28′ E, 39°94′ N). We collected and propagated plants, planted seedling in 20-cm-diameter, 20-cm-deep plastic pots filled with TS1 nutrient medium (Klasmann-Deilmann, Germany). Fresh tissues of *P. alopecuroides* cv. 'Liqiu' (roots, stems, leaves, and spike) were mixed and sampled for PacBio full-length cDNA sequencing. Total RNA was extracted using the Plant RNA Kit (OMEGA, GA, USA, no. R6827-01). The quantity and integrity of RNA samples were assessed in the NanoDrop ND-1000 spectrophotometer (NanoDrop Technologies, DE, USA) and in the 2100 Bioanalyzer (Agilent Technologies, CA, USA), respectively. Qualified RNA samples were then used for constructing PacBio full-length cDNA libraries.

### 2.2 Library construction and genome sequencing

Genomic DNA was extracted by using DNAsecure Plant Kit (Tiangen, Beijing, China) from fresh young leaves of *P. alopecuroides* cv. 'Liqiu' which were harvested and frozen immediately in liquid nitrogen. For Illumina sequencing, a library with an insert size of 350 bp was constructed and sequenced using an Illumina Novaseq 6000 platform (Illumina, San Diego, CA, United States) to produce a short-read sequencing library (Hu et al., 2022). Short reads generated from the Illumina platform were used for the estimation of the genome size, the level of heterozygosity, and the repeat content of the genome, and long reads from the PacBio platform were used for genome assembly. The short-reads from Illumina platform were quality-filtered by HTQC v1.92.310 (Yang et al., 2013) using the following method. First, the adaptors were removed from the sequencing reads. Second, read pairs were excluded if any one end has an average quality lower than 20. Third, the ends of reads were trimmed if the average quality is lower than 20 in the sliding window size of 5 bp. Finally, read pairs with any end that was shorter than 75 bp were removed. The quality-filtered reads were used for genome size estimation. For PacBio sequencing, 8–20-kb fragment library was used for multi-round sequencing of a single fragment to improve the accuracy. The constructed library was sequenced for third-generation sequencing by PacBio Sequencing II platform (Pacific Biosciences of California, Menlo Park, CA, USA). After reversing the crosslinks, we constructed Hi-C fragment libraries from 300- to 700-bp insert size and performed sequencing through the Illumina platform (Rao et al., 2014).

### 2.3 Hi-C technology helps anchor contigs

We constructed Hi-C fragment libraries ranging from 300- to 700-bp insert size as illustrated in Rao et al. (2014) and performed sequencing through the Illumina platform. For anchored contigs, clean read pairs were generated from HiC-Pro v2.10.0 (Servant et al., 2015) and were mapped to the polished genome using BWA v.0.7.10-r789 (Li and Durbin, 2009) with the default parameters. Only uniquely aligned pair reads whose mapping quality was more than 20 were retained for further analysis. Invalid read pairs, including dangling-end and self-cycle, re-ligation, and dumped products, were filtered by HiC-Prov2.10.0. Paired reads with mate mapped to a different contig were used to do the Hi-C-associated scaffolding. Self-ligation, non-ligation, and other invalid reads, such as Start NearRsite, PCR amplification, random break, large and small fragments, and extreme fragments, were filtered (Burton et al., 2013). Then, the contigs were clustered into groups with the agglomerative hierarchical clustering method in Lachesis. Lachesis software (Burton et al., 2013) was further applied to order and orient the clustered contigs. The parameters were CLUSTER_MIN_RE_SITES = 86, CLUSTER_MAX_LINK_DENSITY = 2, ORDER_MIN_N_RES_IN_TRUNK = 72, and ORDER_MIN_N_RES_IN_SHREDS = 69. After Hi-C error correction, redundancy removal, and clustering and ordering the contigs, we obtained the final version of the genome.

### 2.4 Annotation of repetitive sequences

Transposon element (TE) and tandem repeats were annotated by the following workflows. TEs were identified by a combination of homology-based and *de novo* approaches. We first customized a *de novo* repeat library of the genome using RepeatModeler (http://www.repeatmasker.org/RepeatModeler/), which can automatically execute two *de novo* repeat finding programs, including RECON and RepeatScout (Bao and Eddy, 2002; Price et al., 2005). Then, full-length long-terminal repeat retrotransposons (fl-LTR-RTs) were identified using both LTRharvest (v1.5.9) (Ellinghaus et al., 2008) and LTR_finder (v2.8) (Xu and Wang, 2007). The high-quality intact fl-LTR-RTs and non-redundant LTR library were then produced by LTR_retriever (Ou and Jiang, 2018). The non-redundant species-specific TE library was constructed by combining the *de novo* TE sequences library above with the known Dfam (v3.5) database. Final TE sequences in the *P. alopecuroides* genome were identified and classified by homology search against the library using RepeatMasker (v4.12) (Chen, 2004). Tandem repeats were found by Tandem Repeats Finder (TRF 409) (Benson, 1999) and MIcroSAtellite identification tool (MISA v2.1) (Beier et al., 2017).

### 2.5 Gene prediction

We integrated three approaches, namely, *de novo* prediction, homology search, and transcript-based assembly, to annotate protein-coding genes in the genome. The *de novo* gene models were predicted using two *ab initio* gene prediction software tools, Augustus (v3.1.0) (Stanke et al., 2008) and SNAP (2006-07-28) (Korf, 2004). For the homolog-based approach, GeMoMa (v1.7) (Keilwagen et al., 2016) software was employed by using reference gene models from the other six species, which included *O. sativa*, *C. purpureus*, *S. italica*, *A. thaliana*, *C. americanus*, and *S. viridis*. For the transcript-based prediction, RNA sequencing data were mapped to the reference genome using Hisat (v2.1.0) (Kim et al., 2015) and assembled by Stringtie (v 2.1.4) (Pertea et al., 2015). GeneMarkS-T (v5.1) (Tang et al., 2015) was used to predict genes based on the assembled transcripts. The PASA (v2.4.1) (Haas et al., 2003) software was used to predict genes based on the unigenes [and full-length transcripts from the PacBio (ONT) sequencing] assembled by Trinity (v2.11) (Grabherr et al., 2011). Gene models from these different approaches were combined using the EVM software (v1.1.1) (Haas et al., 2008) and updated by PASA.

### 2.6 Annotation of protein-coding genes

Gene functions were inferred according to the best match of the alignments to the National Center for Biotechnology Information (NCBI) Non-redundant (NR) (20200921, ftp://ftp.ncbi.nlm.nih.gov/blast/db), EggNOG (5.0, http://eggnog5.embl.de/download/eggnog_5.0/) (Huerta-Cepas et al., 2019), GO, (20200615, http://geneontology.org), Kyoto Encyclopedia of Genes and Genomes (KEGG) (20191220, http://www.genome.jp/kegg) (Kanehisa et al., 2016) with an E-value threshold of 1E-5, Pfam (v33.1, http://pfam.xfam.org) (Finn et al., 2006), and SwissProt (202005, http://ftp.ebi.ac.uk/pub/databases/swissprot) (Boeckmann et al., 2003).

### 2.7 Annotation of non-coding RNAs

Non-coding RNAs of tRNA were identified using scan-SE (version 1.3.1) (Lowe and Eddy, 1997). Identification of the rRNA genes was conducted by barrnap (v0.9) (Benson, 1999) based on Rfam (v14.5) (Griffiths-Jones et al., 2005). miRNA and snRNA genes were predicted using infernal (v1.1) (Nawrocki and Eddy, 2013) against the Rfam (v14.5) database with default parameters.

### 2.8 Pseudogene prediction

Pseudogenes usually have similar sequences to functional genes but may have lost their biological function because of some genetic mutations, such as insertion and deletion. The GenBlastA (v1.0.4) (She et al., 2009) program was used to scan the whole genomes after masking predicted functional genes. Putative candidates were then analyzed by searching for premature stop mutations and frame-shift mutations using GeneWise (v2.4.1) (Birney et al., 2004).

### 2.9 Detection of methylation sites

*P. alopecuroides* cv. 'Liqiu' was sequenced by PacBio HiFi (CCS), with a total data volume of 41.92 Gb. Based on the quality control of CCS methylation site test data, using SMRTLINK (V11.0) (https://www.pacb.com/support/software-downloads), high-quality CCS reads were obtained. At the same time, kinetic signals were obtained by combining the parameters—all kinetics and kinetic signals in CCS reads were converted into methylation states by the primrose (https://github.com/mattoslmp/primrose) (called 5mC in CpG motifs) program. The 5mC methylation information was stored in the tag tags (MM and ML) of bam files. The CpG/5mC information was analyzed from the above-mentioned data using pb-CpG-tools (v1.0.0) (Ni et al., 2021) software. Each chromosome was divided into multiple Windows with a size of 100 kb, and the average methylation level of each window was calculated.

### 2.10 Phylogenetic tree reconstruction

Using the obtained single-copy gene family sequences, IQ-TREE (v1.6.11) (Nguyen et al., 2015) software was used to construct the evolutionary tree. Specifically, MAFFT (v7.205) (Katoh et al., 2009) was used to align each single-copy gene family sequence, and then PAL2NAL (v14) (Suyama et al., 2006) was used to convert the aligned protein sequence (protein alignment) into codon alignment (codon alignment). Then, Gblocks (v0.91b) (Talavera and Castresana, 2007) (parameter: -b5 = h) was used to remove regions with poor or large sequence alignment. All the well-ordered gene families of each species were connected end to end, and then each species ends up with a supergene end to end. The optimal model is JTT+F+I+G4 based on the model checking tool ModelFinder (Kalyaanamoorthy et al., 2017). Then, the optimal model was used to construct the evolutionary tree through the maximum likelihood (ML) method, where the bootstrap times were set to 1,000. For the above-mentioned evolutionary tree, we set the outgroup as *A. trichopoda* to obtain a rooted tree and then used the software package MCMCTREE that comes with PAML v4.9i (Yang, 1997) software to calculate the divergence time. The sequences of the single-copy gene families of 16 species were aligned, and a rooted tree was obtained. *A. trichopoda* vs. *S. italica* fossils were obtained at 179–199.1 mya, *S. italica* vs. *S. viridis* fossils at 0.68 to 2.11, and *C. americanus* vs. *C. purpureus* at 3.35–5.01 mya, *S. bicolor* vs. *M. lutarioriparius* at 2.88–8.02 mya, and *O. sativa* vs. *S. italica* at 40.3 to 51.9 mya. The divergence times were calculated using the package MCMCTREE in PAML v4.9i software.

### 2.11 Genome-wide replication event analysis

Using diamond v0.9.29.130 (Buchfink et al., 2015), we compared the collinear regions in the genomes of *P. alopecuroides* and *O. sativa* and in *S. viridis* to determine similar gene pairs. MCScanX (Wang et al., 2012) software was used to determine whether similar genes were adjacent on chromosomes based on gene location information files. The proportion of base conversion mutations at the fourfold synonymous (degenerative) third-codon transversion (4DTv) locus in each homologous gene was calculated through https://github.com/JinfengChen/Scripts and then corrected 4DTv using the HKY substitution model to obtain the distribution map. The peak was identified as the whole-genome duplication event in this species.

## 3 Results

### 3.1 Genome assembly, quality validation, and annotation

A 350-bp library was constructed using the genomic DNA of the sample, and 106.72 Gb of high-quality data was obtained by Illumina sequencing platform. The total sequencing depth was approximately ×106.55, and the proportion of Q20 was more than 98.82%. The proportion of Q30 reached 93.49%, and the GC content was approximately 44.78%. After the quality assessment of *P. alopecuroides* genome, the average depth of k-mer constructed was 77 (**Supplementary Figure S1A**). The calculated estimated genome length was approximately 1.00 Gb. According to the distribution of k-mer, the content of repeat sequence was estimated to be approximately 74.46%, and the heterozygosity was estimated to be approximately 0.43%. The data volume of ccs sequencing was 41.92 Gb, and the depth was approximately ×41. The average length of ccs produced by statistics was more than 17,995 bp, and the longest ccs length is 50,190 bp (**Supplementary Table S1**). The maximum number of ccs is around 18,000 bp in length (**Supplementary Table S2**). Using Hifiasm (Cheng et al., 2021) splice, the total length of the contig sequence was 845.71 Mb, the contig N50 was 84.83 Mb, and the scaffold N50 was 91.33 Mb (**Supplementary Table S4**). The CEGMA evaluation completeness was 99.13% (**Supplementary Table S3**). OrthoDB 10 embryophyta database was selected, and the completeness of BUSCO evaluation was 98.45% (**Supplementary Table S3**). The short sequence obtained from the second-generation sequencing data was matched with the assembled genome, and 99.22% of the sequences were matched correctly. The response ratio of the third generation of reads was 98.46%, the coverage was 99.99, and the average sequencing depth was 49 (**Supplementary Figure S1B**).

The Hi-C library was sequenced by the Illumina high-throughput sequencing platform. A total of 125.30 Gb of clean data was generated. A total of 342,693,530 uniquely aligned genomic reads were obtained, of which 142,777,564 were valid Hi-C data, accounting for 41.66% of uniquely aligned genomic data. After Hi-C assembly and manual adjustment of heat map, 833.41 Mb of genome sequence was located on nine chromosomes, accounting for 98.55% (**Figure 1B**). The longest (115.63 Mb) and shortest (74.04 Mb) chromosomes were chr2 and chr6, respectively (**Supplementary Table S5**). The TE density, gene density, and GC content of every chromosome are shown together on a circos plot (**Figure 1C**). The 830.09-Mb sequence that could determine direction and sequence information accounts for 99.6% of the total length of sequences located on chromosomes.

### 3.2 Repeat annotation

We identified a total of 512,992,680 bp TE sequence, which accounted for 60.66% of the genome. The specific non-LTR-RTs mainly consisted of long interspersed nuclear elements (LINEs) and short interspersed nuclear elements (SINE). Based on the analysis of transposon elements in the repetitive sequence in the *P. alopecuroides* cv. 'Liqiu' genome, we found that LINE, SINE, and DNA transposons accounted for 0.92%, 0.13%, and 8.87%, respectively (**Supplementary Table S6**). The LTR-RTs accounted for 50.73%, while the most abundant LTRs were *Copia* elements, making up 30.47% of the genome, followed by *Gypsy* elements (8.47%). About 45,931,763 bp of tandem repeats sequence were obtained, accounting for 5.43% of the genome (**Supplementary Table S7**).

### 3.3 Gene prediction and functional validation

**Figure 2:**
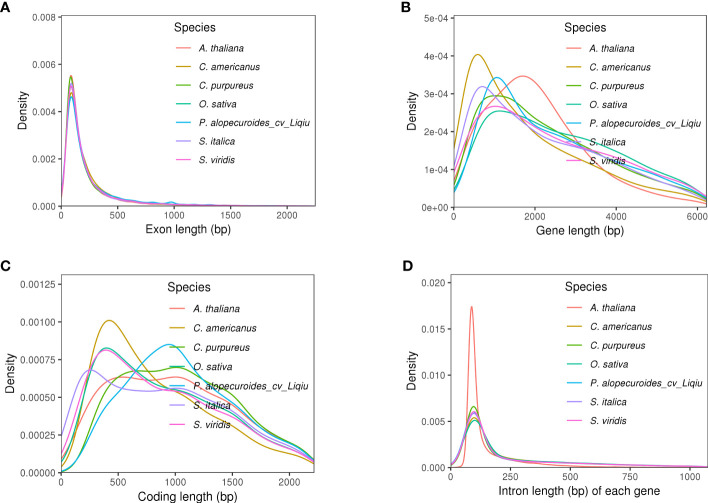
Genetic structural characteristics of genomic prediction. **(A)** Extron length of five species, the trend of distribution of which was similar. **(B)** Gene length of five species, and *P. alopecuroides* had the highest density at around 1,750 bp compared with other plants. **(C)** Coding length of five species. **(D)** Intron length of five species, the trend of distribution of which was similar, and *P. alopecuroides* density was relatively small.

A total of 34,312 genes were obtained by homology, *de novo*, and transcriptome prediction (**Supplementary Figure S2**, **Supplementary Table S8**). The average lengths of the genes and CDS are 1,266.31 and 1,286.37 bp, respectively, and there are 4.95 exons in each gene with a length of 1,400.87 bp per exon (**Figure 2A**, **Supplementary Table S9**). Among them, 19,951 gene models were supported by all three lines of evidence. The genetic characteristics were more closely related to the genetic relations of the species. Therefore, compared with the genetic characteristics of the closely related species, we observed the quality of our prediction. The length of most genes is distributed around 1,600 bp, and that of the coding genes is around 1,000 bp (**Figures 2B, C**; **Supplementary Table S12**). The distribution of intron length and mRNA length is similar to those of other reference grasses (**Figure 2D**). The BUSCO database of embryophyta contains 1,614 conserved core genes. We used BUSCO (v5.2.2) software to evaluate the integrity of gene prediction, of which 98.51% of BUSCO genes existed in the genes, indicating the high integrity of gene prediction. We predicted 824 tRNAs, 7,114 rRNAs, and 233 miRNAs, which were non-coding RNAs. In addition, we predicted 276 pseudogenes. A total of 97.5% of genes were annotated based on the prediction of NR, eggNOG, GO, KEGG, KOG, and Pfam databases (**Supplementary Figure S5**, **Supplementary Table S10**). The highly abundant tandem repeats dispersed on chromosomes with high TE density like chr1, chr2, chr5 and chr7. The distribution of TE density across chromosomes for chr1–6, chr8, and chr9 shows a tendency for higher density in centromeric and less on pericentromeric. However, chr7 density is high at the beginning of the chromosome and then gradually decreases.

### 3.4 Detection of methylation sites

**Figure 3:**
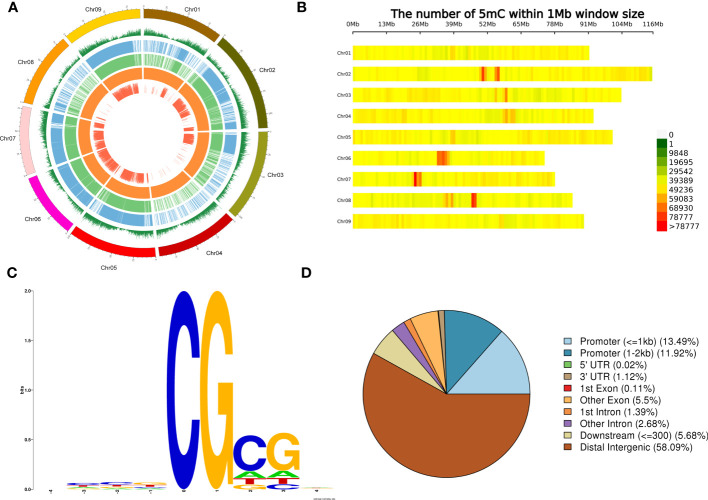
Genome-wide methylation levels and distribution of methylation on chromosomes. **(A)** Genome-wide methylation levels. From outside to inside, respectively, of a chromosome: gene density window (the higher level represented a greater number of genes), level 1 of methylation haplotype, level 2 of methylation, haplotype whole-genome methylation level differentially methylated regions, single size or samples. **(B)** Density distribution of methylation sites on each chromosome according to the 1-Mb window. The horizontal coordinate is chromosome length, and each band represents a chromosome. The genome is divided according to the size of 1 Mb. The more methylation sites in each window, the darker the color, and the fewer methylation sites, the lighter the color. The darker the area in the diagram is the one where the methylation sites are concentrated. **(C)** Nine-base-pair sequence distribution around site 5mC. The horizontal coordinate is the relative position of the methylation site, the total height of each position is the sequence conservation of the base at that position, and the height of the base signal represents the relative frequency of the base at that position. **(D)** Functional annotation and enrichment of a differentially methylated region.

A total of 37,729,477 5mC sites were obtained, among which the number of haplotype 1 and 2 methylation sites was 18,363,029 and 14,959,046, respectively (**Figure 3A**). The distribution of methylation sites across the nine chromosomes was obtained from the average level calculated from the partitioned 100-kb window. The gene density in the window showed high telomere density and low centromere density. The methylation levels and distribution of haplotypes 1 and 2 were similar. The overall methylation level is high. According to the detected methylation sites, chr2, chr6, chr7, and chr8 were found to have concentrated methylation regions in the centromere (**Figure 3B**).

In the 1,000 5mC sites with the highest methylation level, CG was found to be highly conserved in the analysis of the sequence characteristics of the surrounding 9 bp (**Figure 3C**). The differentially methylated regions (DMRs) were annotated to different gene regions, and 58.09% were found in distal intergenic regions. It is followed by promoter (≤1 kb) and promoter (1 to 2 kb) (**Figure 3D**). The DMR annotated to the non-intergenic region of the gene was used for KEGG enrichment. Most of them were enriched to RNA polymerase, followed by tryptophan metabolism (**Supplementary Figure S4**).

### 3.5 Gene family analysis and genome evolution

**Figure 4:**
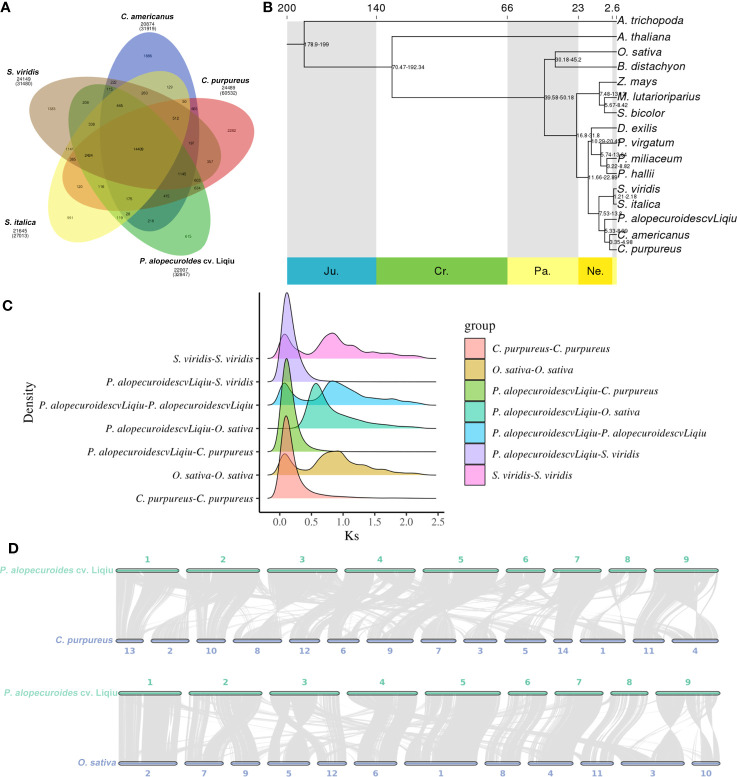
Comparative and evolutionary genomic analysis of *P. alopecuroides*. **(A)** Gene family clustering petal map. The number below the species name is the total number of gene families, the corresponding number of genes is in parentheses, and the number of gene families is in the Venn diagram. **(B)** Evolutionary tree with time for differentiation. **(C)** Distribution of average synonymous substitution levels (Ks) between syntenic blocks. Peaks in maps made by interspecies combinations represent species divergence, and peaks produced by assembly-made maps within species represent genome-wide replication events within the species. **(D)** Linear collinear graph between *P. alopecuroides* vs. *C. purpureu* and *P. alopecuroides* vs. *O. sativa.*.

An analysis of gene families in 16 species was performed and annotated, and finally GO and KEGG enrichment analyses were performed for the gene families unique to this species. We obtained 50,510 gene families, and 2,627 gene families were common families (**Supplementary Figure S5A**). In addition, the gene family information of *P. alopecuroides* cv. 'Liqiu' and *S. viridi*, *S. italica*, *C. purpureus*, and *C. americanus* were analyzed (**Figure 4A**; **Supplementary Figure S5B**). *P. alopecuroides* contains a total of 22,007 gene families and 34,312 genes, among which 615 are unique gene families. The unique gene family was obviously enriched in RNA polymerase (**Supplementary Figure S6**). As for the copy types of genes, the single copy genes accounted for the largest proportion in *P. alopecuroides* (**Supplementary Figure S7**). From these 16 species, we identified 774 one-to-one single-copy genes that were used to construct a maximum likelihood (ML) tree to show the evolutionary relationships (**Figure 4B**; **Supplementary Table S11**). We set the outgroup to *A. trichopoda* and get a rooted tree. According to the phylogenetic tree, *P. alopecuroides* cv. 'Liqiu' separated from Poaceae approximately 7.53–13.8 million years ago, separating from *C. americanus* and *C. purpureus* approximately 5.33–8.99 million years ago. *C. americanus* and *C. purpureu* separated around 3.35–4.98 million years ago.

Through the analysis of expansion and contraction of gene families, it was found that 637 gene families contracted and 223 gene families expanded during the evolution of *P. alopecuroides* (**Supplementary Figure S8**). The expansion and contraction gene families of this species were analyzed by clusterProfile for GO and KEGG enrichment. The GO enrichment analysis revealed that the contracted gene family was significantly enriched in biological processes related to defense response (**Supplementary Figure S9A**), ADP binding (**Supplementary Figure S9B**), and extracellular region (**Supplementary Figure S9C**). The expanded gene family is highly enriched in pathways associated with DNA repair (**Supplementary Figure S9D**), ribosome (**Supplementary Figure S9E**), and DNA helicase activity (**Supplementary Figure S9F**). The KEGG enrichment analysis showed that the contracted gene family was significantly enriched in the biological processes related to flavonoid biosynthesis (**Supplementary Figure S10A**). The expanded gene family was obviously enriched in “tryptophan metabolism” and “ribosome” (**Supplementary Figure S10B**).

### 3.6 Positive selection and collinearity analysis of *P. alopecuroides*

Single-copy gene families in gene family expansion and contraction analysis were extracted, and the CodeML module in PAML was used for positive selection analysis, resulting in 80 positive selection genes in *P. alopecuroides*. The KEGG enrichment analysis of the 80 positively selected genes and the positively selected genes were enriched in “ABC transporters” (**Supplementary Figure S11**).

The GO enrichment analysis mainly divided 264 positive selections into three categories: biological processes, cell components, and molecular functions (**Supplementary Figure S12A**). Among them, some genes in the biological process mainly play a role in “DNA repair” (**Supplementary Figure S12B**). The number of genes in the “ribosome” is the highest in the cellular component (**Supplementary Figure S12C**). The molecular functions mainly focus on enzyme activity, including “DNA helicase activity” and “nucleotide binding” (**Supplementary Figure S12D**).

### 3.7 Whole-genome duplication and collinearity analysis in *P. alopecuroides*

Genome-wide replication events are the major driving force in species divergence. To estimate the timing of WGD events in the *P. alopecuroides* genome, we described synonymous substitution rates at synonymous nucleotide sites (Ks) between *P. alopecuroides* and three other species, including *O. sativa*, *S. viridis*, and *C. purpureus* (**Figure 4C**). The peaks generated by combinatorial mapping within species represent genome-wide duplication events in this species, which occurred twice during the evolution of *P. alopecuroides*. They occurred when Ks was 0.85 and 0.10, respectively. According to the differentiation time calculation formula: time = Ks/2*μ*, *μ* = 6.1 × 10^–9^. *P. alopecuroides* was separated from *O. sativa* after the first WGD event and before the most recent. Similarly, *O. sativa*, *S. viridis*, and *P. alopecuroides* have similar peaks, indicating that they both experienced two WGD events and that they occurred at similar times. *C. purpureus* had only one WGD event. The evolutionary process of *S. viridis* was closer to *P. alopecuroides*. These two genome-wide duplication events occurred approximately 69.67 and 8.20 mya. We confirmed that two WGD events had occurred during the evolution of *P. alopecuroides* based on 4DTv method (**Supplementary Figure S13A**).

The collinearity of chromosomes was obtained by mapping the sequences of *C. americanus*, *C. purpureus*, *O. sativa*, and *S. viridis* to *P. alopecuroides* chromosomes (**Figure 4D**, **Supplementary Figure S13B**). *C. purpureus* had the highest collinearity ratio with *P. alopecuroides* (63.12%) and the highest number of collinearity genes (62,987). The number of collinear genes of *S. viridis* was 40,048 (55.42%). After screening, the number of collinearity blocks in non-major corresponding chromosomes was less than 100, and the linear collinearity between *P. alopecuroides* and the four species was obtained. The corresponding collinear gene numbers were as follows: *C. americanus*—170, *C. purpureus*—250, *O. sativa*—925, and *S. viridis*—242. Based on JCVI-filtered information (Tang et al., 2008), the distribution of collinear genes on the chromosomes was provided. Among the collinearity of *S. viridis* and *P. alopecuroides*, chr2, chr5, chr6, chr7, chr8, and chr9 were observed to have a large collinear block, and the chromosomes were highly conserved. chr1, chr3, and chr4 undergo chromosomal rearrangement events. In comparison with rice, all the genetic information of chr7 chromosome comes from chr4 of rice. chr2 is mainly the fusion of chr7 and chr9 of rice, chr3 is mainly the fusion of chr5 and chr12, and chr5 is mainly the fusion of chr1 and chr5.

### 3.8 Analysis of the LTR insertion time

**Figure 5:**
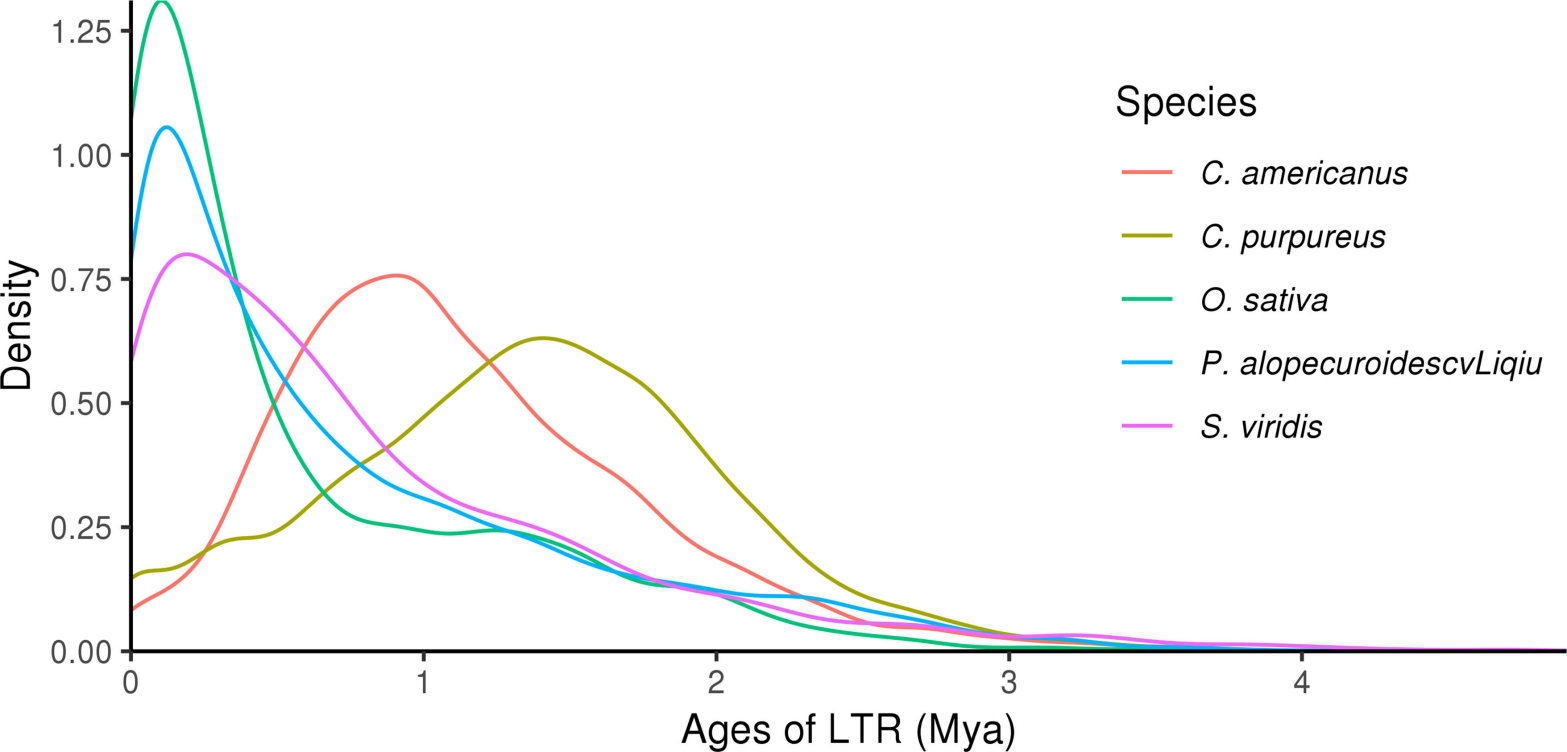
Long terminal repeat insertion time analysis.

The analysis of LTR insertion time of five species showed that the insertion time of LTR-RTs in *P. alopecuroides* was different from that in the other four species, but three of them were relatively late. The analysis of insertion time suggested that LTR insertion was a continuous process and that continuous insertion of LTR may be a general phenomenon in angiosperms (**Figure 5**). The proliferation of LTR-RTs in *P. alopecuroides* (~4.54 mya) and *O. sativa* (~4.08 mya) was earlier than in *S. viridis* (~7.16 mya) but later than in *C. purpureus* (~54.23 mya) and *C. americanus* (~34.79 mya). We observed that LTR insertion was a continuous process that surges at some time, which may have negatively affected the growth of grasslands and other types of vegetation during the Pleistocene when cold weather and limited global atmospheric CO_2_ (180 ppm) were present. To adapt to the stressed abiotic environment, most plants activate TEs to cope with environmental stress. These conditions may have led to plant genome recombination during this period. The insertion time of LTR also spikes after the recombination event in plants (Huang et al., 2019). These results suggest that most of the LTR-RTs in these four genomes were recently inserted, and these insertions occurred well after the divergence of these species.

## 4 Discussion

*Pennisetum* ecological grasses are increasingly popular because of their elegant ecology form, strong stress resistance, and low maintenance. However, due to the complex genomes of these grasses with high heterozygosity and high amounts of repetitive sequences, there is currently a lack of reference genomes, which hinders the genetic studies of these grasses. Here we assembled the *P. alopecuroides* cv. 'Liqiu' genome at the chromosomal level with a size of approximately 845.71 Mb, contig N50 of 84.83 Mb, and genome integrity of 99.13% as assessed by CEGMA. The N50 of this reference genome (contig N50 84.83 Mb) was much higher than that of forage perennial ryegrass genome (contig N50 1,637 kb). Meanwhile, it is better than other sequenced genomes of grasses such as *P. purpureus* (Zhang et al., 2022a), *C*. *purpureus* (contig N50 of 1.83 Mb) (Yan et al., 2020), *C. americanus* (contig N50 of 1.82 kb) (Varshney et al., 2017), *Dactylis glomerata* L. (contig N50 of 0.93 Mb) (Huang et al., 2019), and other grasses such as *Achnatherum splendens* (contig N50 of 40.30 Mb) (Ren et al., 2022) and *Eremochloa ophiuroides* (contig N50 of 40.30 Mb) (Wang et al., 2021). A total of 833.41 Mb sequences were located on nine chromosomes by Hi-C technology.

Polyploidy or whole-genome duplication events provided the original genetic material for biological evolution, and duplication of plant genomes would be extremely important for plant evolution with rapid recombination, loss of a large number of genes, and increase of structural variation (D'Hont et al., 2012; Shi et al., 2022). The early WGD events that the Gramineae collectively experienced, approximately 60 mya, marked the end of the Cretaceous period (Jiao et al., 2014). The replication genes retained after this stage are associated with heat and cold stress, osmotic pressure maintenance, salt stress, water acquisition, and wound repair (Jiao et al., 2014; Gao et al., 2018). The genome analysis showed that the last WGD event occurred in *P. alopecuroides* after differentiation from other monocotyledonous plants, and the corresponding family and genus had already differentiated, which was estimated to have occurred at around 8.2 mya. Ancient chromosome reconstruction of grasses revealed 12 chromosomes after two WGD events and rearrangement. We also found that *P. alopecuroides* and *Setaria* have similar evolutionary characteristics, providing important sources of genetic, evolutionary, and breeding information.

In addition to polyploidization, TE/LTR activity is an important mechanism for karyotype and genome size evolution in angiosperms (Bennetzen et al., 2005), and it is involved in stress responses and probably important for adaptive evolution in dynamically changing environments (VanBuren et al., 2015). The insertion of LTR-RTs is a common phenomenon in angiosperms, and its number reaches a peak between 0 and 1 mya in the Pleistocene (or ice) age (Huang et al., 2019). In this study, LTR insertion was observed to be a continuous process. Previous studies have shown that TE activity in rice increases the total number of stress-induced genes to help rice adapt to stress (Lisch, 2013). Most terrestrial plants and green algae based on the gypsy type of LTR (Jiao et al., 2020), such as Ty1/*Copia* and Ty3/*Gypsy*, occupied 8.43% and 30.47%, respectively, in the *E. ophiuroides* genome which has 867.43 Mb (Wang et al., 2021) and 7.61% and 22.66% in the hawthorn genome with 823.41 Mb (Zhang et al., 2022b). In *P. alopecuroides*, the proportion of Ty1/*Copia* family was 8.43%, indicating that the Ty1/*Copia* family contributes more to the genomic expansion of *P. alopecuroides* than the Ty3/*Gypsy* family. In addition, we consider that LTR was a major cause of genomic expansion, and differences in LTR activity types and timing of divergence may have led to new functions for *P. alopecuroides* (Jiao et al., 2020). The peak of LTR insertion occurred after the recent WGD event, indicating that the LTR insertion could cooperate with WGD events for chromosome deletion, ectopia, and other rearrangements, thus affecting the *P. alopecuroides* genome size and promoting the adaptation of species to the environment.

## 5 Conclusion

We constructed a comprehensive and complete high-quality *P. alopecuroides* genome for the first time, which sheds new light on genetic evolution and polyploid breeding of *P. alopecuroides*. We assembled an 833.41-Mb genome located on nine chromosomes, with 84.83-Mb contig N50, higher than the assembly data for other grasses, to provide a reference for the assembly and annotation of grass plants. The phylogenetic analysis revealed that *P. alopecuroides* was a sister group of *Cenchrus* L., and the proliferation of LTR‐RTs and WGD event analysis verified that two WGD events occurred in *P. alopecuroides*, which provided a reference genome for *Pennisetum* and laid the foundation for analyzing the molecular mechanism of high yield and stress resistance and mining the functional genes related to the target traits.

## Data availability statement

The data presented in the study are deposited in the NCBI repository, accession number PRJNA952524.

## Author contributions

YY and XF conceived the study and designed the experiments. JW and WT collected the experimental materials. YG and LL performed genome analyses. KT and QG completed the manuscript with suggestions from HZ, HW, CZ, XH, and YX. All authors contributed to the article and approved the submitted version.

## References

[B1] Badouin H. Gouzy J. Grassa C. J. Murat F. Staton S. E. Cottret L.. (2017). The sunflower genome provides insights into oil metabolism, flowering and Asterid evolution. Nature 546 (7656), 148–152. doi: 10.1038/nature22380 28538728

[B2] Baek S. Choi K. Kim G.-B. Yu H.-J. Cho A. Jang H.. (2018). Draft genome sequence of wild Prunus yedoensis reveals massive inter-specific hybridization between sympatric flowering cherries. Genome Biol. 19 (1), 127. doi: 10.1186/s13059-018-1497-y PMC6124018 30180884

[B3] Bao Z. Eddy S. R. (2002). Automated *de novo* identification of repeat sequence families in sequenced genomes. Genome Res. 12 (8), 1269–1276. doi: 10.1101/gr.88502 12176934 PMC186642

[B4] Beier S. Thiel T. Münch T. Scholz U. Mascher M. (2017). MISA-web: a web server for microsatellite prediction. Bioinformatics 33 (16), 2583–2585. doi: 10.1093/bioinformatics/btx198 28398459 PMC5870701

[B5] Bennetzen J. L. Ma J. Devos K. M. (2005). Mechanisms of recent genome size variation in flowering plants. Ann. Bot. 95 (1), 127–132. doi: 10.1093/aob/mci008 15596462 PMC4246713

[B6] Bennetzen J. L. Schmutz J. Wang H. Percifield R. Hawkins J. Pontaroli A. C.. (2012). Reference genome sequence of the model plant Setaria. Nat. Biotechnol. 30 (6), 555–561. doi: 10.1038/nbt.2196 22580951

[B7] Benson G. (1999). Tandem repeats finder: a program to analyze DNA sequences. Nucleic Acids Res. 27 (2), 573–580. doi: 10.1093/nar/27.2.573 9862982 PMC148217

[B8] Birney E. Clamp M. Durbin R. (2004). GeneWise and genomewise. Genome Res. 14 (5), 988–995. doi: 10.1101/gr.1865504 15123596 PMC479130

[B9] Boeckmann B. Bairoch A. Apweiler R. Blatter M. C. Estreicher A. Gasteiger E.. (2003). The SWISS-PROT protein knowledgebase and its supplement TrEMBL in 2003. Nucleic Acids Res. 31 (1), 365–370. doi: 10.1093/nar/gkg095 12520024 PMC165542

[B10] Buchfink B. Xie C. Huson D. H. (2015). Fast and sensitive protein alignment using DIAMOND. Nat. Methods 12 (1), 59–60. doi: 10.1038/nmeth.3176 25402007

[B11] Burton J. N. Adey A. Patwardhan R. P. Qiu R. Kitzman J. O. Shendure J. (2013). Chromosome-scale scaffolding of *de novo* genome assemblies based on chromatin interactions. Nat. Biotechnol. 31 (12), 1119–1125. doi: 10.1038/nbt.2727 24185095 PMC4117202

[B12] Byrne S. L. Nagy I. Pfeifer M. Armstead I. Swain S. Studer B.. (2015). A synteny-based draft genome sequence of the forage grassLolium perenne. Plant J. 84 (4), 816–826. doi: 10.1111/tpj.13037 26408275

[B13] Cai J Liu X Vanneste K Proost S Liu ZJ. (2015). The genome sequence of the orchid Phalaenopsis equestris. Nature Genetics 47, 304–304. 10.1038/ng0315-304a 25711871

[B14] Chen N. (2004). Using RepeatMasker to identify repetitive elements in genomic sequences. Curr. Protoc. Bioinf. doi: 10.1002/0471250953.bi0410s05. Chapter 4, Unit 4.10. 18428725

[B15] Cheng H. Concepcion G. T. Feng X. Zhang H. Li H. (2021). Haplotype-resolved *de novo* assembly using phased assembly graphs with hifiasm. Nat. Methods 18 (2), 170–175. doi: 10.1038/s41592-020-01056-5 33526886 PMC7961889

[B16] D'Hont A. Denoeud F. Aury J. M. Baurens F. C. Carreel F. Garsmeur O.. (2012). The banana (Musa acuminata) genome and the evolution of monocotyledonous plants. Nature 488 (7410), 213–217. doi: 10.1038/nature11241 22801500

[B17] Dong S. Liu M. Liu Y. Chen F. Yang T. Chen L.. (2021). The genome of Magnolia biondii Pamp. provides insights into the evolution of Magnoliales and biosynthesis of terpenoids. Hortic. Res. 8 (1), 38. doi: 10.1038/s41438-021-00471-9 33642574 PMC7917104

[B18] Dudhate A. Shinde H. Yu P. Tsugama D. Takano T. (2021). Comprehensive analysis of NAC transcription factor family uncovers drought and salinity stress response in pearl millet (Pennisetum glaucum). BMC Genomics 22 (1), 70. doi: 10.1186/s12864-021-07382-y 33478383 PMC7818933

[B19] Ellinghaus D. Kurtz S. Willhoeft U. (2008). LTRharvest, an efficient and flexible software for *de novo* detection of LTR retrotransposons. BMC Bioinf. 9, 18. doi: 10.1186/1471-2105-9-18 PMC2253517 18194517

[B20] Finn R. D. Mistry J. Schuster-Böckler B. Griffiths-Jones S. Hollich V. Lassmann T.. (2006). Pfam: clans, web tools and services. Nucleic Acids Res. 34 (Database issue), D247–D251. doi: 10.1093/nar/gkj149 16381856 PMC1347511

[B21] Gallego L. J. Escobar A. Penuela M. Pena J. D. Rios L. A. (2015). King Grass: A promising material for the production of second-generation butanol. Fuel 143, 399–403. doi: 10.1016/j.fuel.2014.11.077

[B22] Gao B. Chen M. Li X. Liang Y. Zhu F. Liu T.. (2018). Evolution by duplication: paleopolyploidy events in plants reconstructed by deciphering the evolutionary history of VOZ transcription factors. BMC Plant Biol. 18 (1), 256. doi: 10.1186/s12870-018-1437-8 30367626 PMC6204039

[B23] Grabherr M. G. Haas B. J. Yassour M. Levin J. Z. Thompson D. A. Amit I.. (2011). Full-length transcriptome assembly from RNA-Seq data without a reference genome. Nat. Biotechnol. 29 (7), 644–652. doi: 10.1038/nbt.1883 21572440 PMC3571712

[B24] Griffiths-Jones S. Moxon S. Marshall M. Khanna A. Eddy S. R. Bateman A. (2005). Rfam: annotating non-coding RNAs in complete genomes. Nucleic Acids Res. 33 (Database issue), D121–D124. doi: 10.1093/nar/gki081 15608160 PMC540035

[B25] Guo Y. D. Liu L. Y. Yue Y. S. Fan X. F. Teng W. J. Zhang H.. (2022). Development of SSR markers based on transcriptome sequencing and verification of their conservation across species of ornamental pennisetum rich. (Poaceae). Agronomy-Basel 12 (7). doi: 10.3390/agronomy12071683

[B26] Haas B. J. Delcher A. L. Mount S. M. Wortman J. R. Smith R. K. Jr. Hannick L. I.. (2003). Improving the Arabidopsis genome annotation using maximal transcript alignment assemblies. Nucleic Acids Res. 31 (19), 5654–5666. doi: 10.1093/nar/gkg770 14500829 PMC206470

[B27] Haas B. J. Salzberg S. L. Zhu W. Pertea M. Allen J. E. Orvis J.. (2008). Automated eukaryotic gene structure annotation using EVidenceModeler and the Program to Assemble Spliced Alignments. Genome Biol. 9 (1), R7. doi: 10.1186/gb-2008-9-1-r7 18190707 PMC2395244

[B28] Hayat K. J. F. S. (2020). Combating soil salinity with combining saline agriculture and phytOmanagement with salt-accumulating plants. Crit. Rev. Environ. Sci. Technol. 50 (7a12). doi: 10.1080/10643389.2019.1646087

[B29] Hou X. Teng K. Guo Q. Zhao C. Gao K. Yue Y.. (2022). Research advances in forage pennisetum resource. Chin. Bull. Bot. 57 (06), 814–825. doi: 10.11983/CBB22195

[B30] Hu G. Cheng L. Cheng Y. Mao W. Qiao Y. Lan Y. (2022). Pan-genome analysis of three main Chinese chestnut varieties. Front. Plant Sci. 13. doi: 10.3389/fpls.2022.916550 PMC9358723 35958219

[B31] Huang L. Feng G. Yan H. Zhang Z. Bushman B. S. Wang J.. (2019). Genome assembly provides insights into the genome evolution and flowering regulation of orchardgrass. Plant Biotechnol. J. 18 (2), 373–388. doi: 10.1111/pbi.13205 31276273 PMC6953241

[B32] Huerta-Cepas J. Szklarczyk D. Heller D. Hernández-Plaza A. Forslund S. K. Cook H.. (2019). eggNOG 5.0: a hierarchical, functionally and phylogenetically annotated orthology resource based on 5090 organisms and 2502 viruses. Nucleic Acids Res. 47 (D1), D309–d314. doi: 10.1093/nar/gky1085 30418610 PMC6324079

[B33] International Brachypodium, I (2010). Genome sequencing and analysis of the model grass Brachypodium distachyon. Nature 463 (7282), 763–768. doi: 10.1038/nature08747 20148030

[B34] Jiao Y. Li J. Tang H. Paterson A. H. (2014). Integrated syntenic and phylogenomic analyses reveal an ancient genome duplication in monocots. Plant Cell 26 (7), 2792–2802. doi: 10.1105/tpc.114.127597 25082857 PMC4145114

[B35] Jiao C. Sørensen I. Sun X. Sun H. Behar H. Alseekh S.. (2020). The penium margaritaceum genome: hallmarks of the origins of land plants. Cell 181 (5), 1097–1111.e1012. doi: 10.1016/j.cell.2020.04.019 32442406

[B36] Kalyaanamoorthy S. Minh B. Q. Wong T. K. F. von Haeseler A. Jermiin L. S. (2017). ModelFinder: fast model selection for accurate phylogenetic estimates. Nat. Methods 14 (6), 587–589. doi: 10.1038/nmeth.4285 28481363 PMC5453245

[B37] Kanehisa M. Sato Y. Kawashima M. Furumichi M. Tanabe M. (2016). KEGG as a reference resource for gene and protein annotation. Nucleic Acids Res. 44 (D1), D457–D462. doi: 10.1093/nar/gkv1070 26476454 PMC4702792

[B38] Katoh K. Asimenos G. Toh H. (2009). Multiple alignment of DNA sequences with MAFFT. Methods Mol. Biol. 537, 39–64. doi: 10.1007/978-1-59745-251-9_3 19378139

[B39] Keilwagen J. Wenk M. Erickson J. L. Schattat M. H. Grau J. Hartung F. (2016). Using intron position conservation for homology-based gene prediction. Nucleic Acids Res. 44 (9), e89. doi: 10.1093/nar/gkw092 26893356 PMC4872089

[B40] Kim D. Langmead B. Salzberg S. L. (2015). HISAT: a fast spliced aligner with low memory requirements. Nat. Methods 12 (4), 357–360. doi: 10.1038/nmeth.3317 25751142 PMC4655817

[B41] Korf I. (2004). Gene finding in novel genomes. BMC Bioinf. 5, 59. doi: 10.1186/1471-2105-5-59 PMC421630 15144565

[B42] Li H. Durbin R. (2009). Fast and accurate short read alignment with Burrows-Wheeler transform. Bioinformatics 25 (14), 1754–1760. doi: 10.1093/bioinformatics/btp324 19451168 PMC2705234

[B43] Liang Y. Xiao W. Hui L. Yang T. Lian J. Yang R.. (2015). The genome of dendrobium officinale illuminates the biology of the important traditional chinese orchid herb. Mol. Plant 8 (006), 922–934. doi: 10.1016/j.molp.2014.12.011 25825286

[B44] Lisch D. (2013). How important are transposons for plant evolution? Nat. Rev. Genet. 14 (1), 49–61. doi: 10.1038/nrg3374 23247435

[B45] Liu L. Y. Teng K. Fan X. F. Han C. Zhang H. Wu J. Y.. (2022). Combination analysis of single-molecule long-read and Illumina sequencing provides insights into the anthocyanin accumulation mechanism in an ornamental grass, Pennisetum setaceum cv. Rubrum. Plant Mol. Biol. 109 (1-2), 159–175. doi: 10.1007/s11103-022-01264-x 35338443

[B46] Lovell J. T. Jenkins J. Lowry D. B. Mamidi S. Sreedasyam A. Weng X.. (2018). The genomic landscape of molecular responses to natural drought stress in Panicum hallii. Nat. Commun. 9 (1), 5213. doi: 10.1038/s41467-018-07669-x 30523281 PMC6283873

[B47] Lowe T. M. Eddy S. R. (1997). tRNAscan-SE: a program for improved detection of transfer RNA genes in genomic sequence. Nucleic Acids Res. 25 (5), 955–964. doi: 10.1093/nar/25.5.955 9023104 PMC146525

[B48] Mamidi S. Healey A. Huang P. Grimwood J. Jenkins J. Barry K.. (2020). A genome resource for green millet Setaria viridis enables discovery of agronomically valuable loci. Nat. Biotechnol. 38 (10), 1203–1210. doi: 10.1038/s41587-020-0681-2 33020633 PMC7536120

[B49] Marks R. A. Hotaling S. Frandsen P. B. Vanburen R. (2021). Representation and participation across 20 years of plant genome sequencing. Nat. Plants 7, 1571–1578. doi: 10.1038/s41477-021-01031-8 34845350 PMC8677620

[B50] Mitros T. Session A. M. James B. T. Wu G. A. Belaffif M. B. Clark L. V.. (2020). Genome biology of the paleotetraploid perennial biomass crop Miscanthus. Nat. Commun. 11 (1), 5442. doi: 10.1038/s41467-020-18923-6 33116128 PMC7595124

[B51] Nawrocki E. P. Eddy S. R. (2013). Infernal 1.1: 100-fold faster RNA homology searches. Bioinformatics 29 (22), 2933–2935. doi: 10.1093/bioinformatics/btt509 24008419 PMC3810854

[B52] Nguyen L. T. Schmidt H. A. von Haeseler A. Minh B. Q. (2015). IQ-TREE: a fast and effective stochastic algorithm for estimating maximum-likelihood phylogenies. Mol. Biol. Evol. 32 (1), 268–274. doi: 10.1093/molbev/msu300 25371430 PMC4271533

[B53] Ni P. Huang N. Nie F. Zhang J. Zhang Z. Wu B.. (2021). Genome-wide detection of cytosine methylations in plant from Nanopore data using deep learning. Nat. Commun. 12 (1), 5976. doi: 10.1038/s41467-021-26278-9 34645826 PMC8514461

[B54] Ou S. Jiang N. (2018). LTR_retriever: A highly accurate and sensitive program for identification of long terminal repeat retrotransposons. Plant Physiol. 176 (2), 1410–1422. doi: 10.1104/pp.17.01310 29233850 PMC5813529

[B55] Pertea M. Pertea G. M. Antonescu C. M. Chang T. C. Mendell J. T. Salzberg S. L. (2015). StringTie enables improved reconstruction of a transcriptome from RNA-seq reads. Nat. Biotechnol. 33 (3), 290–295. doi: 10.1038/nbt.3122 25690850 PMC4643835

[B56] Price A. L. Jones N. C. Pevzner P. A. (2005). *De novo* identification of repeat families in large genomes. Bioinformatics 21 (Suppl 1), i351–i358. doi: 10.1093/bioinformatics/bti1018 15961478

[B57] Rao S. S. Huntley M. H. Durand N. C. Stamenova E. K. Bochkov I. D. Robinson J. T.. (2014). A 3D map of the human genome at kilobase resolution reveals principles of chromatin looping. Cell 159 (7), 1665–1680. doi: 10.1016/j.cell.2014.11.021 25497547 PMC5635824

[B58] Ren G. Jiang Y. Li A. Yin M. Li M. Mu W.. (2022). The genome sequence provides insights into salt tolerance of Achnatherum splendens (Gramineae), a constructive species of alkaline grassland. Plant Biotechnol. J. 20 (1), 116–128. doi: 10.1111/pbi.13699 34487631 PMC8710827

[B59] Servant N. Varoquaux N. Lajoie B. R. Viara E. Chen C. J. Vert J. P.. (2015). HiC-Pro: an optimized and flexible pipeline for Hi-C data processing. Genome Biol. 16, 259. doi: 10.1186/s13059-015-0831-x 26619908 PMC4665391

[B60] She R. Chu J. S. Wang K. Pei J. Chen N. (2009). GenBlastA: enabling BLAST to identify homologous gene sequences. Genome Res. 19 (1), 143–149. doi: 10.1101/gr.082081.108 18838612 PMC2612959

[B61] Shi T. Huneau C. Zhang Y. Li Y. Chen J. Salse J.. (2022). The slow-evolving Acorus tatarinowii genome sheds light on ancestral monocot evolution. Nat. Plants 8 (7), 764–777. doi: 10.1038/s41477-022-01187-x 35835857 PMC9300462

[B62] Song C. Liu Y. Song A. Dong G. Chen S. (2018). The Chrysanthemum nankingense Genome Provides Insights into the Evolution and Diversification of Chrysanthemum Flowers and Medicinal Traits. Mol. Plant 11 (12), 1–10. doi: 10.1016/j.molp.2018.10.003 30342096

[B63] Stanke M. Diekhans M. Baertsch R. Haussler D. (2008). Using native and syntenically mapped cDNA alignments to improve *de novo* gene finding. Bioinformatics 24 (5), 637–644. doi: 10.1093/bioinformatics/btn013 18218656

[B64] Sun M. Yan H. Zhang A. Jin Y. Lin C. Lou L. (2023). Milletdb: a multi-omics database to accelerate the research of functional genomics and molecular breeding of millets. Plant Biotechnol. J., 1–10. doi: 10.1111/pbi.14136 PMC10579705 37530223

[B65] Suyama M. Torrents D. Bork P. (2006). PAL2NAL: robust conversion of protein sequence alignments into the corresponding codon alignments. Nucleic Acids Res. 34 (Web Server issue), W609–W612. doi: 10.1093/nar/gkl315 16845082 PMC1538804

[B66] Talavera G. Castresana J. (2007). Improvement of phylogenies after removing divergent and ambiguously aligned blocks from protein sequence alignments. Syst. Biol. 56 (4), 564–577. doi: 10.1080/10635150701472164 17654362

[B67] Tang H. Bowers J. E. Wang X. Ming R. Alam M. Paterson A. H. (2008). Synteny and collinearity in plant genomes. Science 320 (5875), 486–488. doi: 10.1126/science.1153917 18436778

[B68] Tang S. Lomsadze A. Borodovsky M. (2015). Identification of protein coding regions in RNA transcripts. Nucleic Acids Res. 43 (12), e78. doi: 10.1093/nar/gkv227 25870408 PMC4499116

[B69] Tenorio C. Roque R. M. (2015). Quality of pellets made from agricultural and forestry crops in Costa Rican tropical climates. BioResources 10 (1), 482–498. doi: 10.1016/j.molp.2018.10.003

[B70] VanBuren R. Bryant D. Edger P. P. Tang H. Burgess D. Challabathula D.. (2015). Single-molecule sequencing of the desiccation-tolerant grass Oropetium thomaeum. Nature 527 (7579), 508–511. doi: 10.1038/nature15714 26560029

[B71] Varshney R. K. Shi C. Thudi M. Mariac C. Wallace J. Qi P.. (2017). Pearl millet genome sequence provides a resource to improve agronomic traits in arid environments. Nat. Biotechnol. 35 (10), 969–976. doi: 10.1038/nbt.3943 28922347 PMC6871012

[B72] Wang Y. Tang H. Debarry J. D. Tan X. Li J. Wang X.. (2012). MCScanX: a toolkit for detection and evolutionary analysis of gene synteny and collinearity. Nucleic Acids Res. 40 (7), e49. doi: 10.1093/nar/gkr1293 22217600 PMC3326336

[B73] Wang J. Zi H. Wang R. Liu J. Wang H. Chen R.. (2021). A high-quality chromosome-scale assembly of the centipedegrass [Eremochloa ophiuroides (Munro) Hack.] genome provides insights into chromosomal structural evolution and prostrate growth habit. Hortic. Res. 8 (1), 201. doi: 10.1038/s41438-021-00636-6 34465733 PMC8408263

[B74] Xu J. Liu C. Song Y. Li M. (2021). Comparative analysis of the chloroplast genome for four pennisetum species: molecular structure and phylogenetic relationships. Front. Genet. 12. doi: 10.3389/fgene.2021.687844 PMC8354216 34386040

[B75] Xu Z. Wang H. (2007). LTR_FINDER: an efficient tool for the prediction of full-length LTR retrotransposons. Nucleic Acids Res. 35 (Web Server issue), W265–W268. doi: 10.1093/nar/gkm286 17485477 PMC1933203

[B76] Yan H. Sun M. Zhang Z. Jin Y. Zhang A. Lin C.. (2023). Pangenomic analysis identifies structural variation associated with heat tolerance in pearl millet. Nat. Genet. 55 (3), 507–518. doi: 10.1038/s41588-023-01302-4 36864101 PMC10011142

[B77] Yan Q. Wu F. Xu P. Sun Z. Li J. Gao L.. (2020). The elephant grass (Cenchrus purpureus) genome provides insights into anthocyanidin accumulation and fast growth. Mol. Ecol. Resour. 21 (2), 526–542. doi: 10.1111/1755-0998.13271 33040437 PMC7821259

[B78] Yang Z. (1997). PAML: a program package for phylogenetic analysis by maximum likelihood. Comput. Appl. Biosci. 13 (5), 555–556. doi: 10.1093/bioinformatics/13.5.555 9367129

[B79] Yang X. Liu D. Liu F. Wu J. Zou J. Xiao X.. (2013). HTQC: a fast quality control toolkit for Illumina sequencing data. BMC Bioinf. 14(1), 33. doi: 10.1186/1471-2105-14-33 PMC3571943 23363224

[B80] Yue Y. Fan X. Hu Y. Han C. Li H. Teng W.. (2020). *In vitro* induction and characterization of hexaploid Pennisetum × advena, an ornamental grass. Plant C 142 (2), 221–228. doi: 10.1007/s11240-020-01814-5

[B81] Zhang T. Qiao Q. Du X. Zhang X. Hou Y. Wei X.. (2022b). Cultivated hawthorn (Crataegus pinnatifida var. major) genome sheds light on the evolution of Maleae (apple tribe). J. Integr. Plant Biol. 64 (8), 1487–1501. doi: 10.1111/jipb.13318 35748532

[B82] Zhang S. Xia Z. Li C. Wang X. Lu X. Zhang W.. (2022a). Chromosome-scale genome assembly provides insights into speciation of allotetraploid and massive biomass accumulation of elephant grass (Pennisetum purpureum Schum.). Mol. Ecol. Resour 22 (6), 2363–2378. doi: 10.1111/1755-0998.13612 35347881

[B83] Zhang Y. Yuan X. H. Teng W. J. Chen C. Wu J. Y. (2016). Identification and phylogenetic classification of pennisetum (Poaceae) ornamental grasses based on SSR locus polymorphisms. Plant Mol. Biol. Rep. 34 (6), 1181–1192. doi: 10.1007/s11105-016-0990-2

[B84] Zheng T. Li P. Li L. Zhang Q. (2021). Research advances in and prospects of ornamental plant genomics. Horticult Res. 8 (1), 65 doi: 10.1038/s41438-021-00499-x PMC8012582 33790259

