# Supplementary material for: Chromosome-level reference genome assembly provides insights into the evolution of Pennisetum alopecuroides
Source: Front Plant Sci. 2023 Aug 23;14:1195479. doi: 10.3389/fpls.2023.1195479 (PMC10481962; doi:10.3389/fpls.2023.1195479)
Supplement: Supplementary file 1 [file DataSheet_1.pdf]

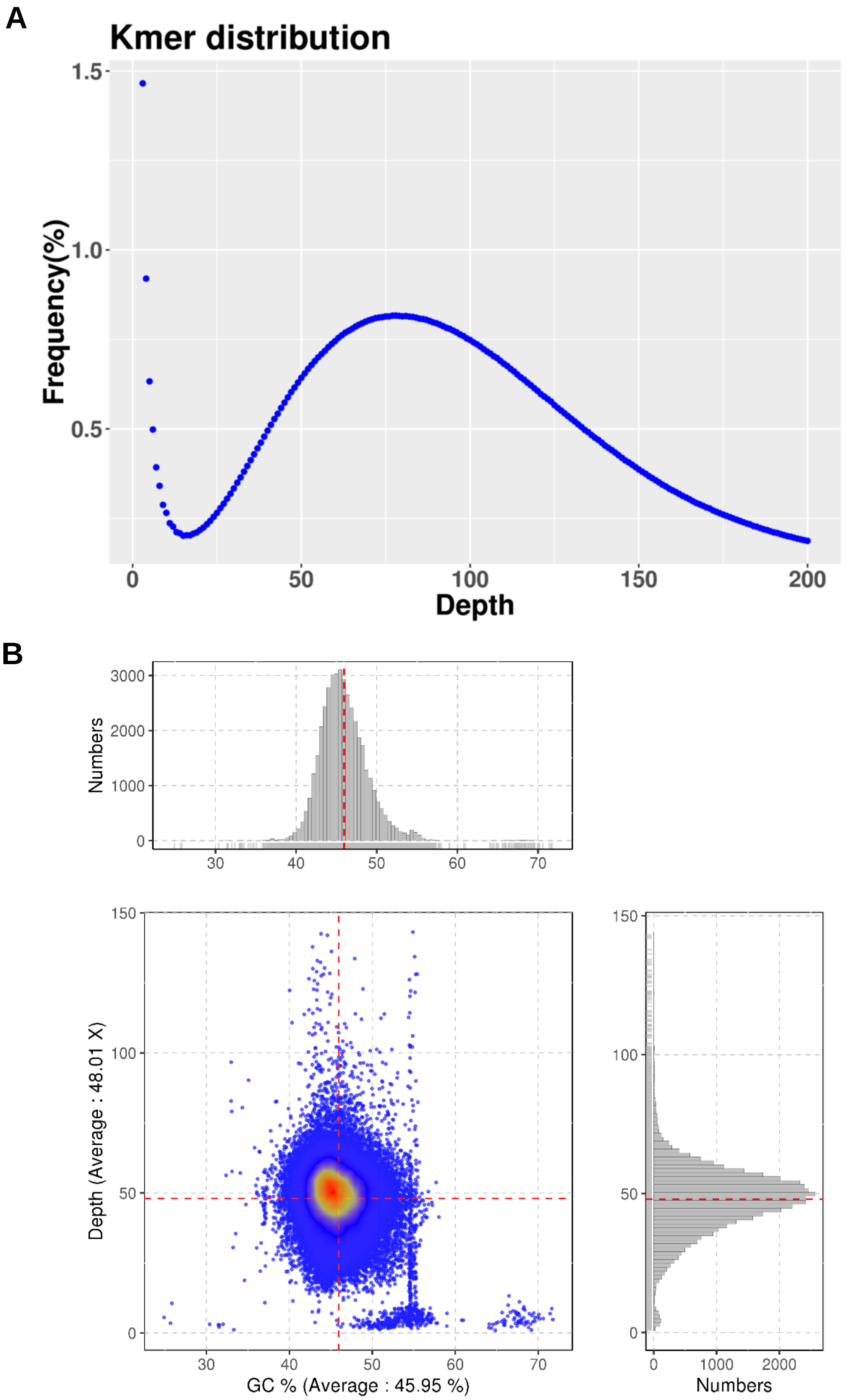

Figure S1 Genome survey information of *P. alopecuroides*. (A) Frequency distribution of depth of in genome survey of *P. alopecuroides* . **(B)** GC depth distribution of *P. alopecuroides* survey. The abscess is GC content, and the ordinate is sequencing depth. The two values were sequentially counted in a 10 Kb window.
