# Supplementary figures and images for: Chromosome-level reference genome assembly provides insights into the evolution of Pennisetum alopecuroides
Source: Front Plant Sci. 2023 Aug 23;14:1195479. doi: 10.3389/fpls.2023.1195479 (PMC10481962; doi:10.3389/fpls.2023.1195479)

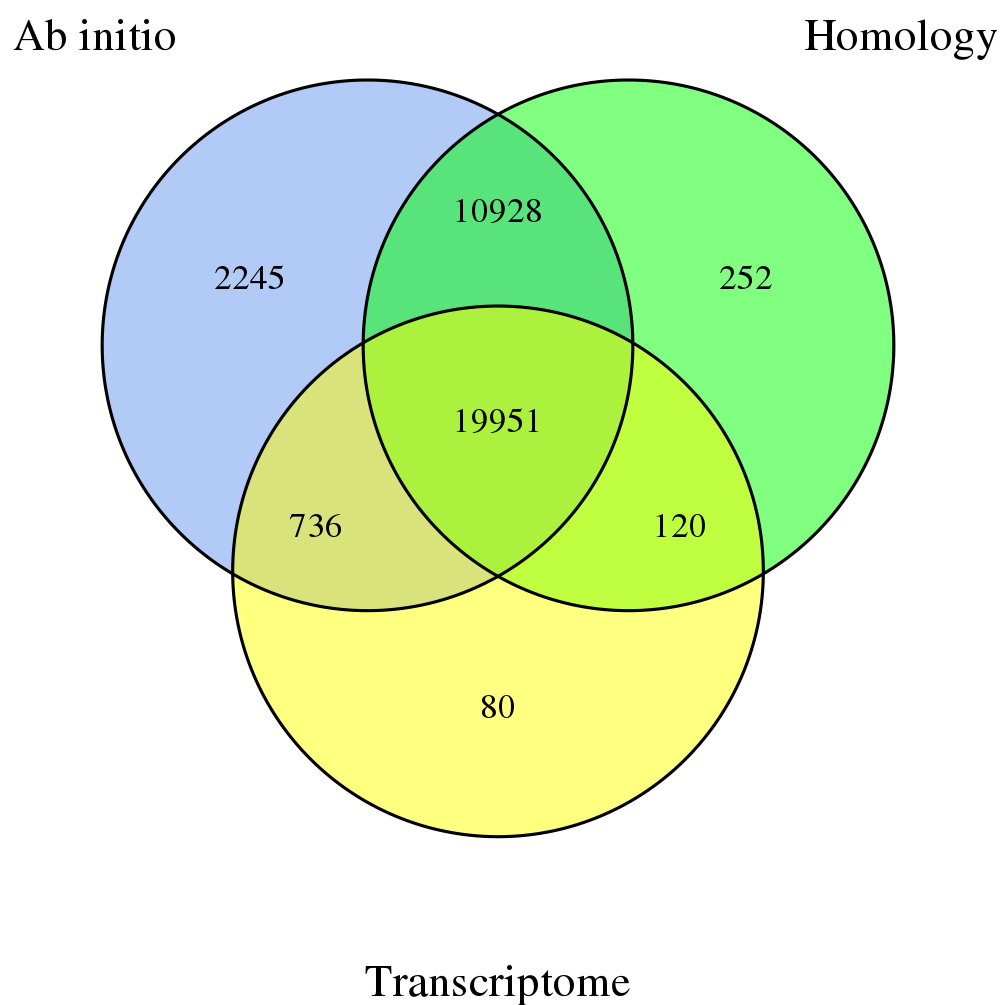

**Figure S2 Integrated gene distribution maps derived from three prediction methods.**

Supplement: Supplementary file 2 [file DataSheet_2.pdf]

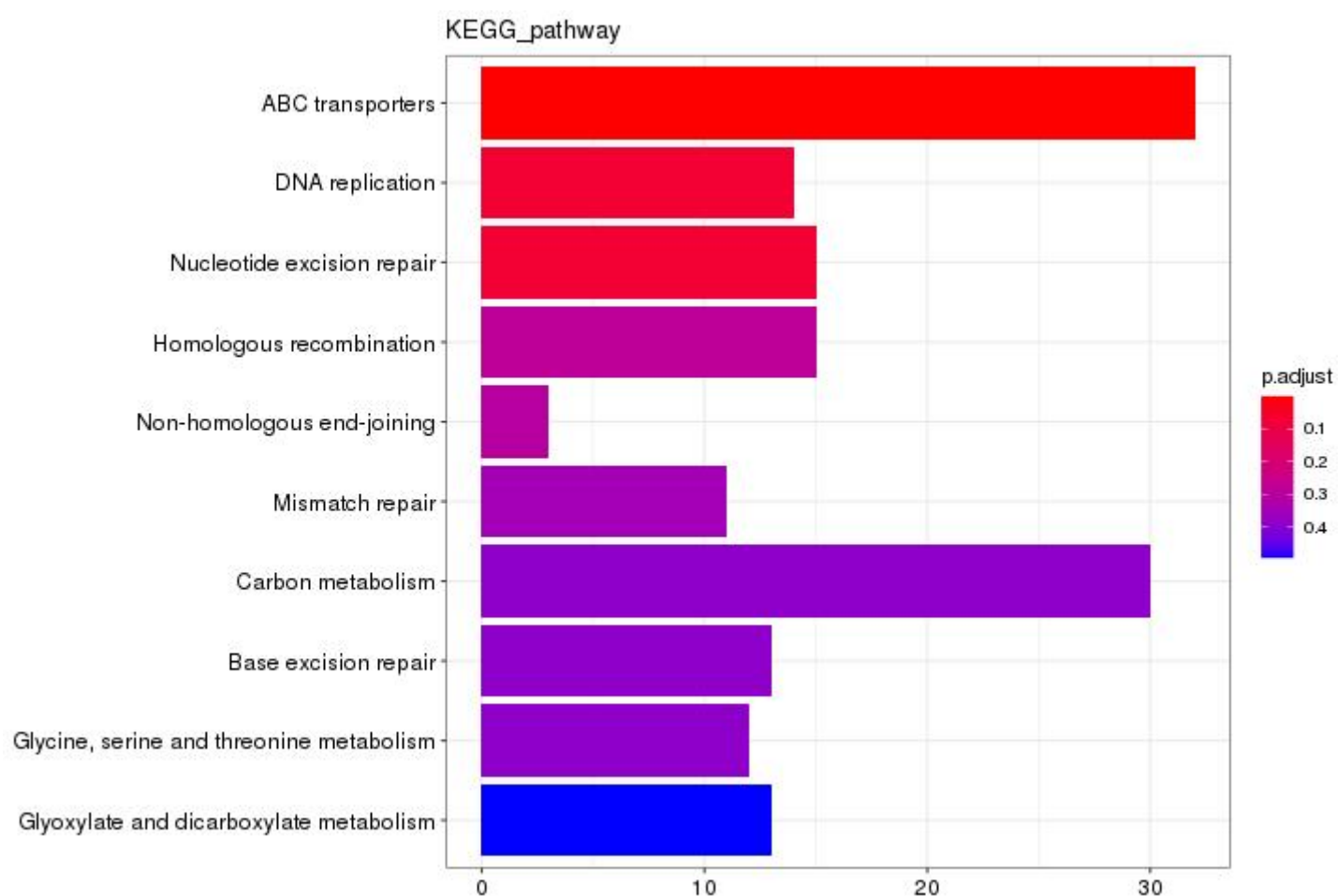

**Figure S4 KEGG enrichment map of DMR associated gene**

Supplement: Supplementary file 4 [file DataSheet_4.pdf]

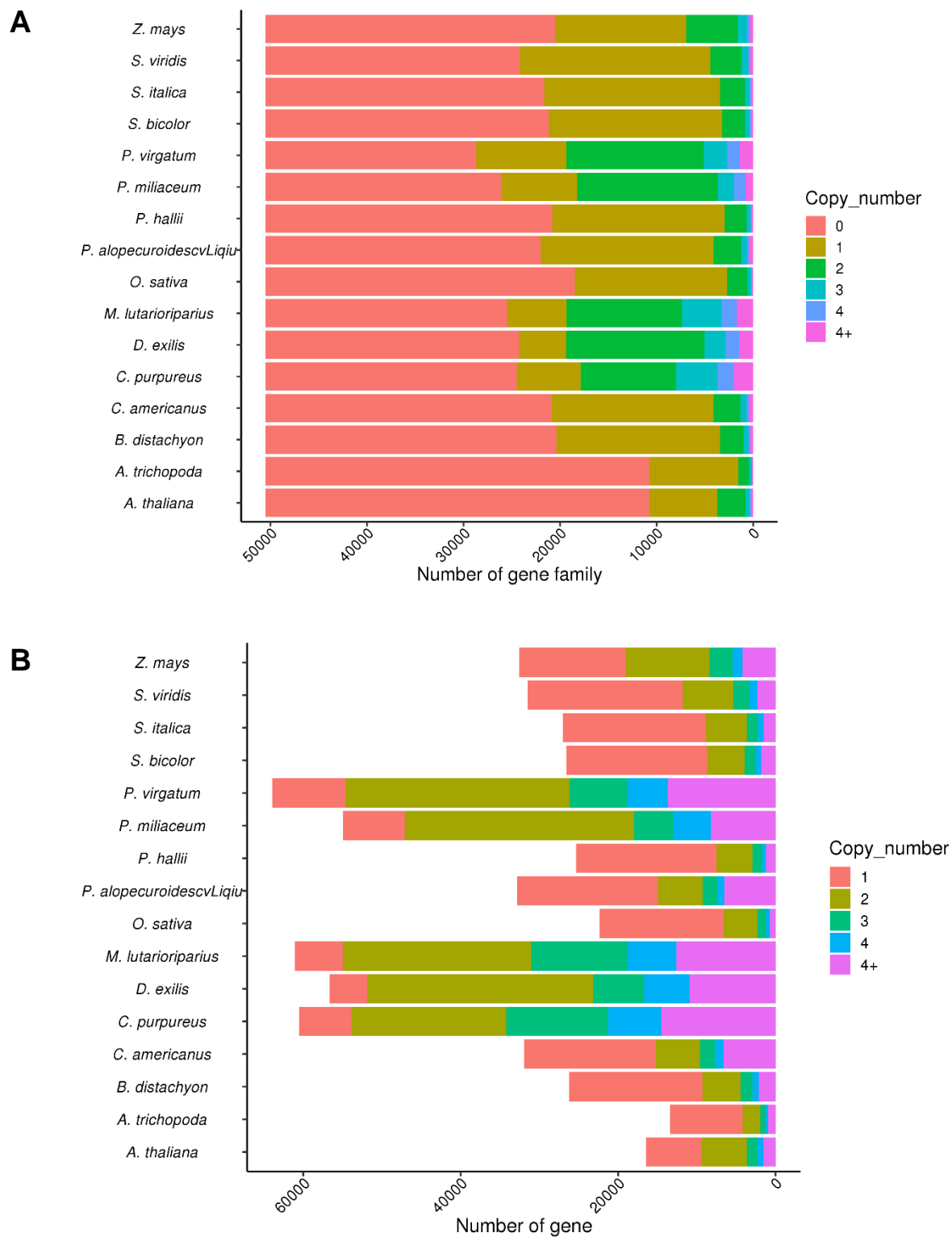

Supplement: Supplementary file 7 [file DataSheet_7.pdf]

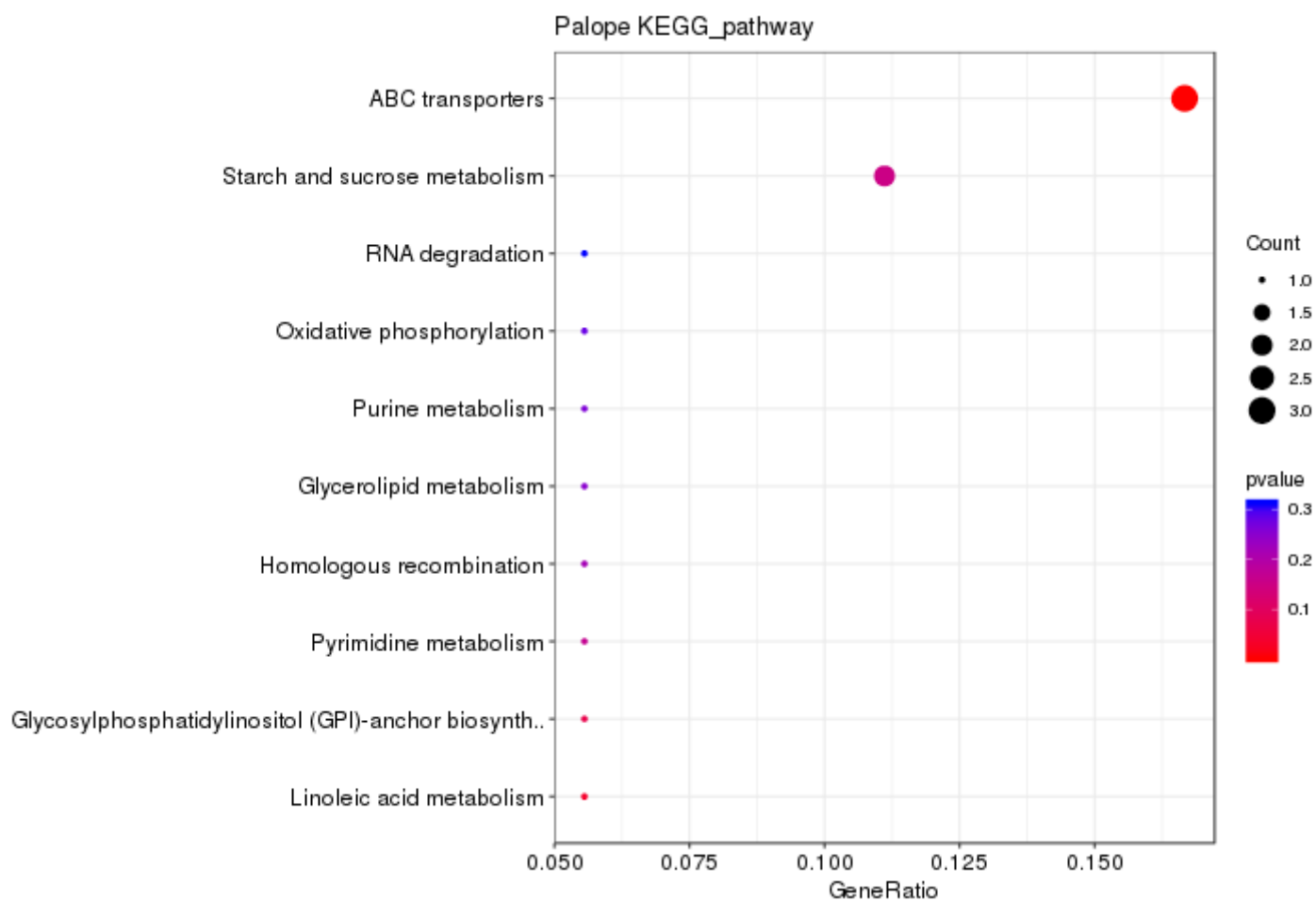

**Figure S11 KEGG enrichment analysis of positive selected genes.**

Supplement: Supplementary file 11 [file DataSheet_11.pdf]
