# Supplementary material for: Chromosome-level reference genome assembly provides insights into the evolution of Pennisetum alopecuroides
Source: Front Plant Sci. 2023 Aug 23;14:1195479. doi: 10.3389/fpls.2023.1195479 (PMC10481962; doi:10.3389/fpls.2023.1195479)
Supplement: Supplementary file 3 [file DataSheet_3.pdf]

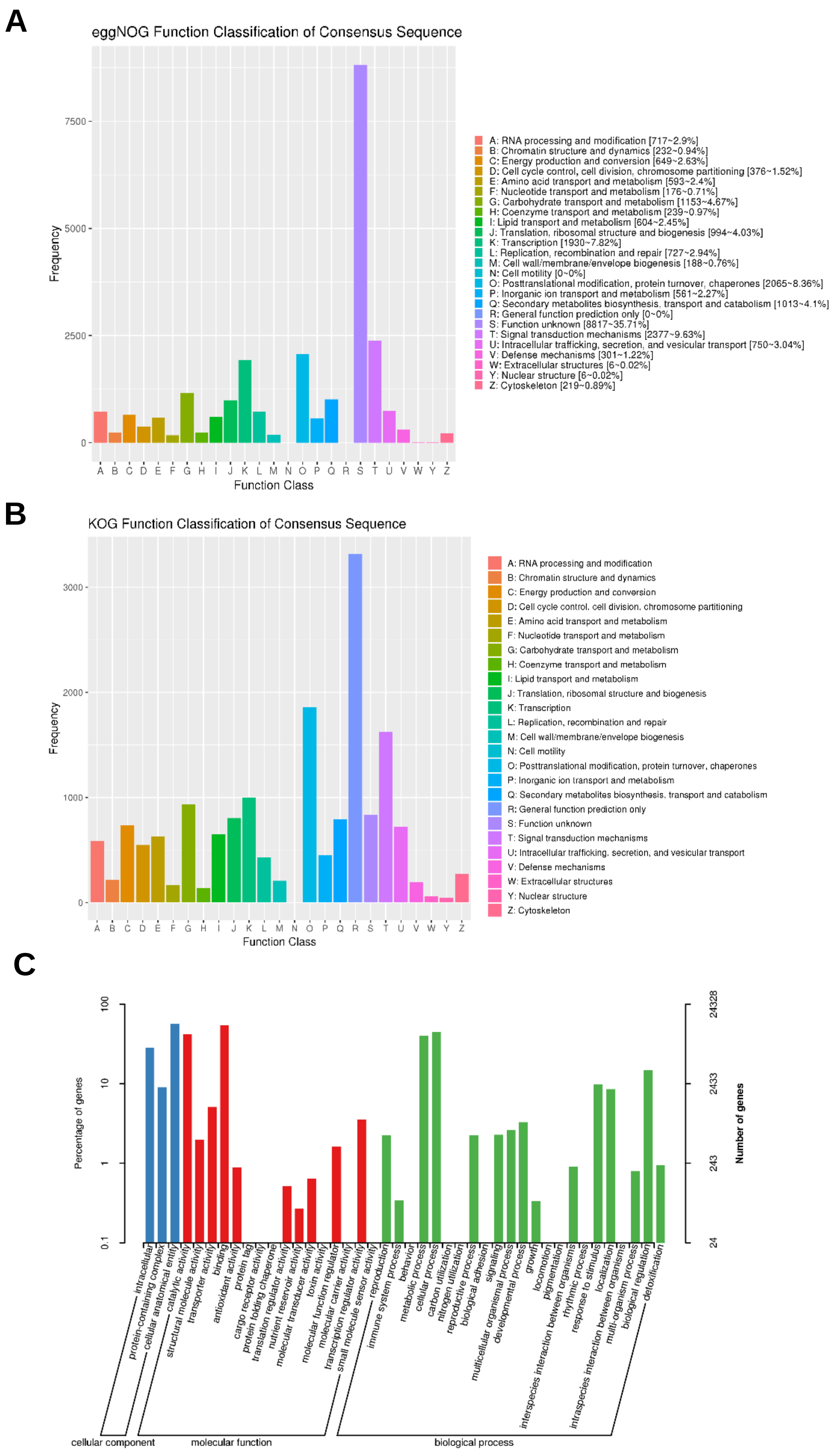

Figure S3 The genome annotation of *P. alopecuroides* based on different databases. (A) eggNOG function annotation classification statistics chart. **(B)** KOG function annotation classification statistics chart. **(C)** GO secondary node annotation classification statistics chart.
