# Supplementary material for: Chromosome-level reference genome assembly provides insights into the evolution of Pennisetum alopecuroides
Source: Front Plant Sci. 2023 Aug 23;14:1195479. doi: 10.3389/fpls.2023.1195479 (PMC10481962; doi:10.3389/fpls.2023.1195479)
Supplement: Supplementary file 5 [file DataSheet_5.pdf]

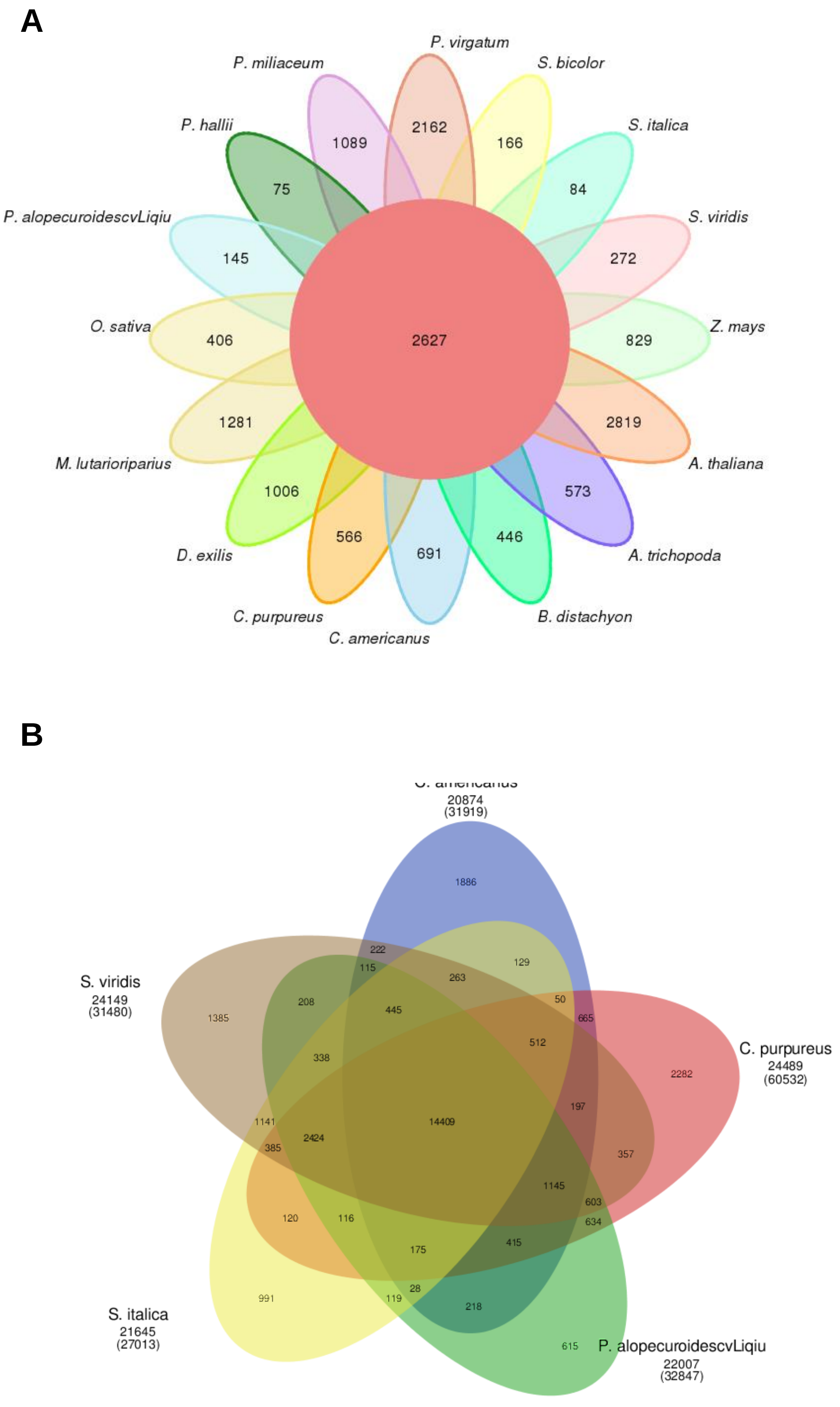

Figure S5 Gene family clustering petal map. (A) The middle circle is the number of gene families shared by all species, and the edge is the number of gene families unique to each species. **(B)** Venn diagram of gene family clustering. The number below the species name is the total number of gene families, the corresponding number of genes in parentheses, and the number of gene families in the Venn diagram.
