# Supplementary material for: Chromosome-level reference genome assembly provides insights into the evolution of Pennisetum alopecuroides
Source: Front Plant Sci. 2023 Aug 23;14:1195479. doi: 10.3389/fpls.2023.1195479 (PMC10481962; doi:10.3389/fpls.2023.1195479)
Supplement: Supplementary file 6 [file DataSheet_6.pdf]

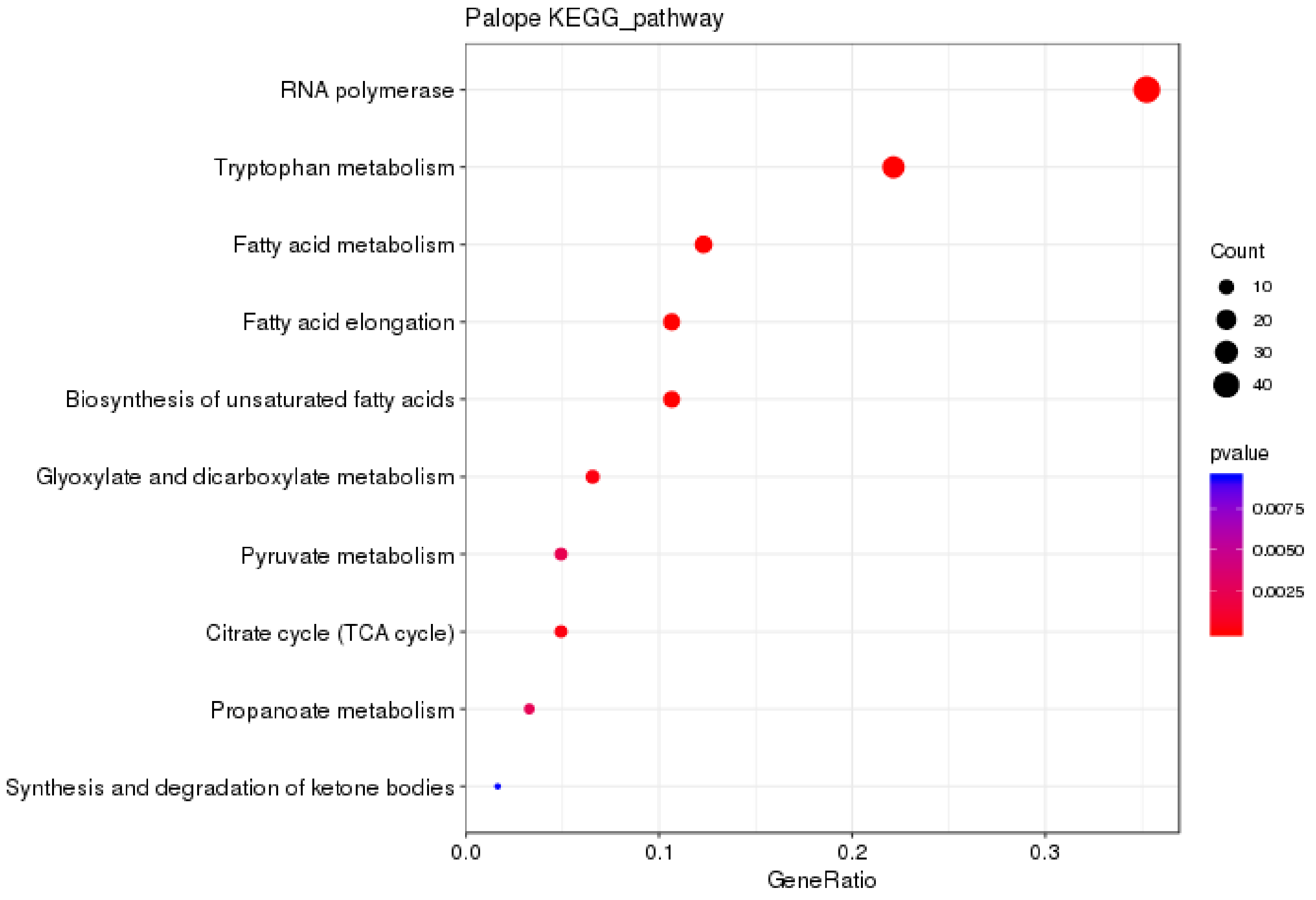

Figure S6 KEGG enrichment analysis of endemic gene families. In the dot plot, the x-coordinate represents the proportion of unique genes under this pathway to the total unique genes. The size of dot represents the number of genes enriched on this pathway, and the color is the corresponding p value.
