# Supplementary material for: Chromosome-level reference genome assembly provides insights into the evolution of Pennisetum alopecuroides
Source: Front Plant Sci. 2023 Aug 23;14:1195479. doi: 10.3389/fpls.2023.1195479 (PMC10481962; doi:10.3389/fpls.2023.1195479)
Supplement: Supplementary file 8 [file DataSheet_8.pdf]

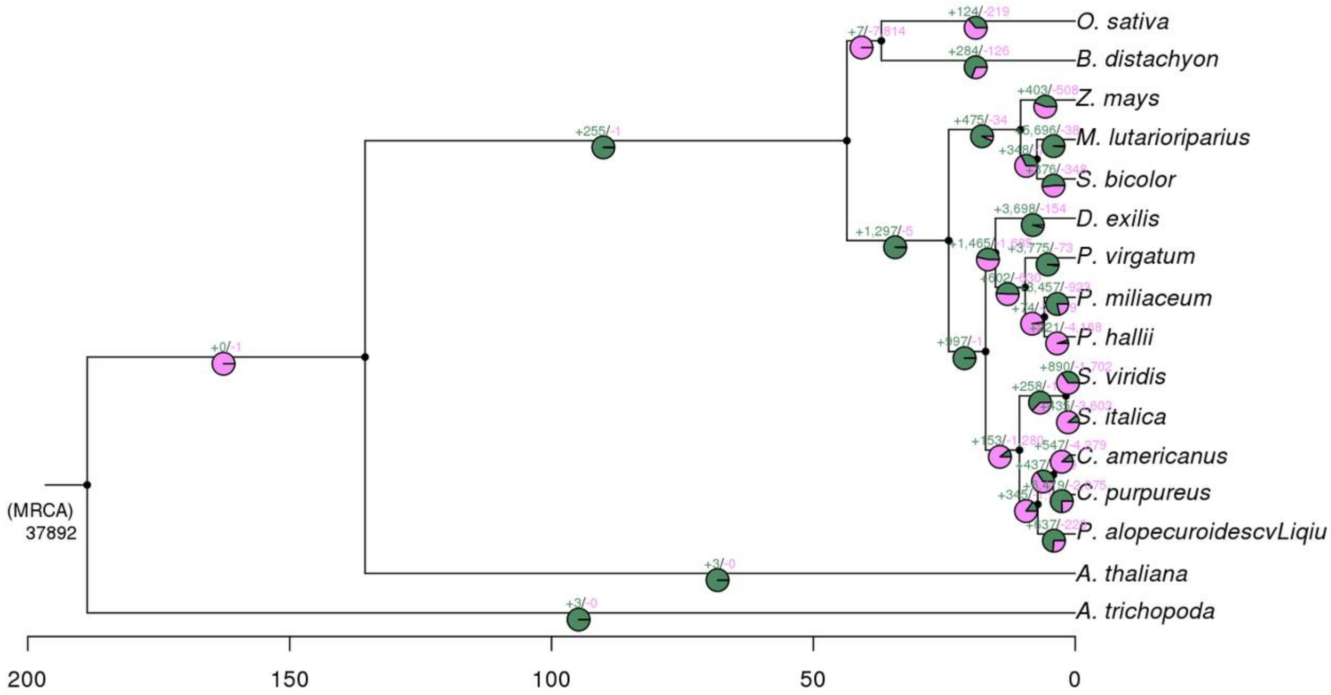

Figure S8 The Maximum likelihood phylogenetic tree of ten selected species and the number of expansion/contraction families in the selected species.
