# Supplementary material for: Chromosome-level reference genome assembly provides insights into the evolution of Pennisetum alopecuroides
Source: Front Plant Sci. 2023 Aug 23;14:1195479. doi: 10.3389/fpls.2023.1195479 (PMC10481962; doi:10.3389/fpls.2023.1195479)
Supplement: Supplementary file 9 [file DataSheet_9.pdf]

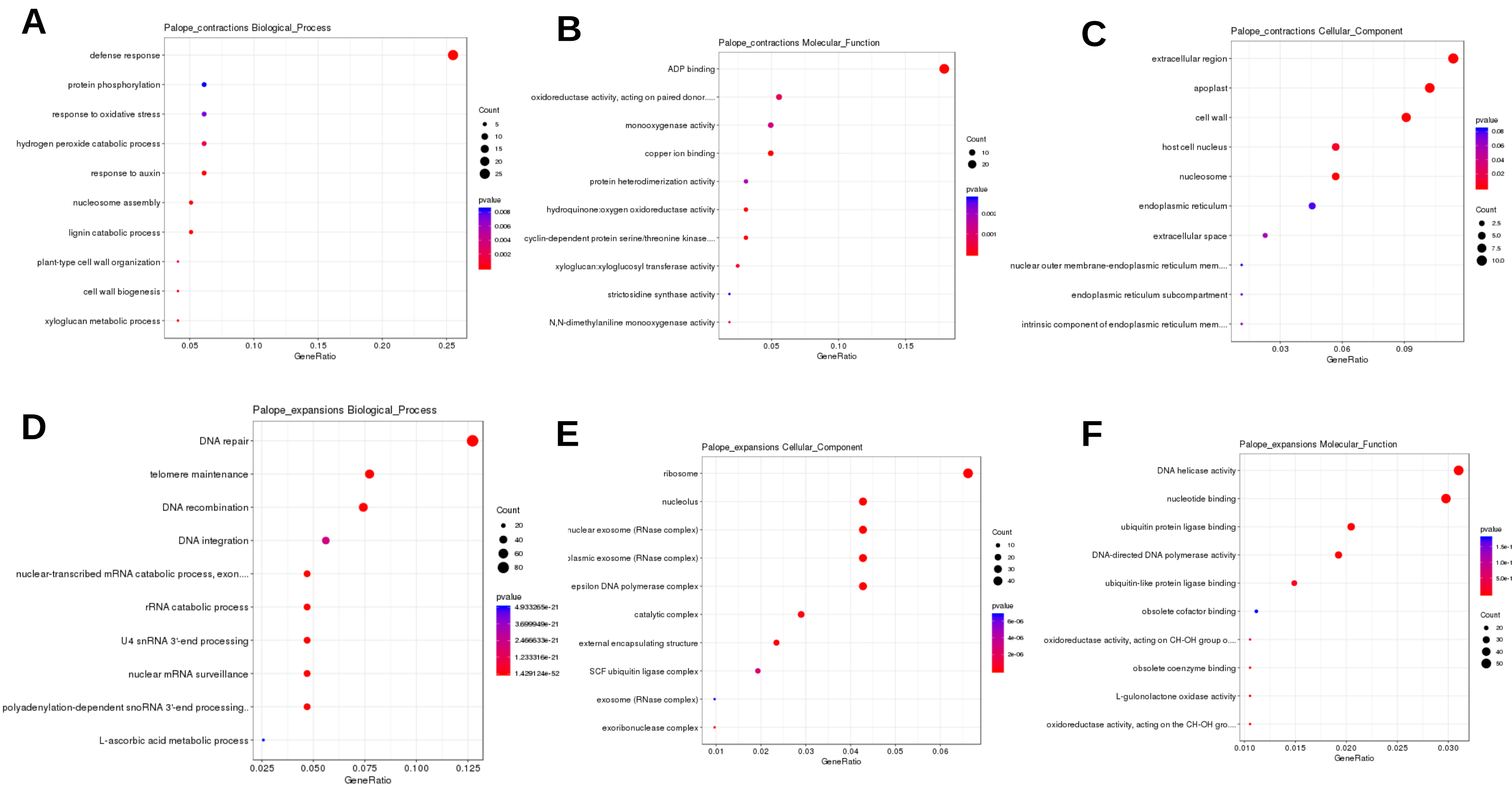


**Figure S9 Enrichment analysis of gene family of *P. alopecuroides*. (A)**The proportion of endemic genes to total contracted gene families under biological process. **(B)** The proportion of endemic genes to contracted gene families under cellular component. **(C) C**ontracted gene families pathways enriched in molecular function. **(D)** The proportion of endemic genes to expended families under biological process. **(E)** The proportion of endemic genes to total expended families under cellular component. **(F)** Expended families p athways enriched in molecular function.
