# Supplementary material for: Chromosome-level reference genome assembly provides insights into the evolution of Pennisetum alopecuroides
Source: Front Plant Sci. 2023 Aug 23;14:1195479. doi: 10.3389/fpls.2023.1195479 (PMC10481962; doi:10.3389/fpls.2023.1195479)
Supplement: Supplementary file 10 [file DataSheet_10.pdf]

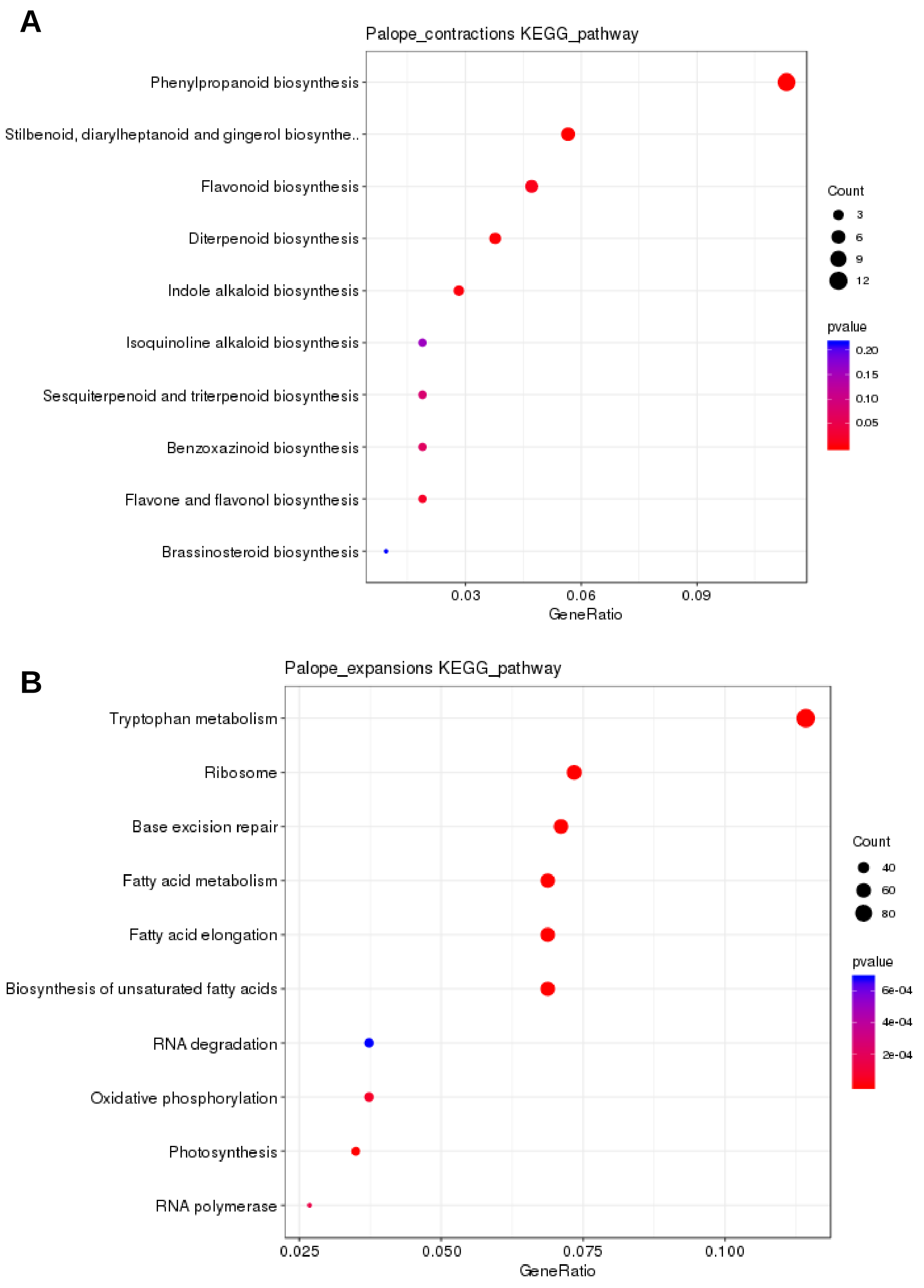

Figure S10 KEGG enrichment analysis of the gene family of *P. alopecuroides*. (A) KEGG enriched pathway of expansion gene family. **(B)** KEGG enriched pathway of contraction gene family.
