# Supplementary material for: Chromosome-level reference genome assembly provides insights into the evolution of Pennisetum alopecuroides
Source: Front Plant Sci. 2023 Aug 23;14:1195479. doi: 10.3389/fpls.2023.1195479 (PMC10481962; doi:10.3389/fpls.2023.1195479)
Supplement: Supplementary file 12 [file DataSheet_12.pdf]

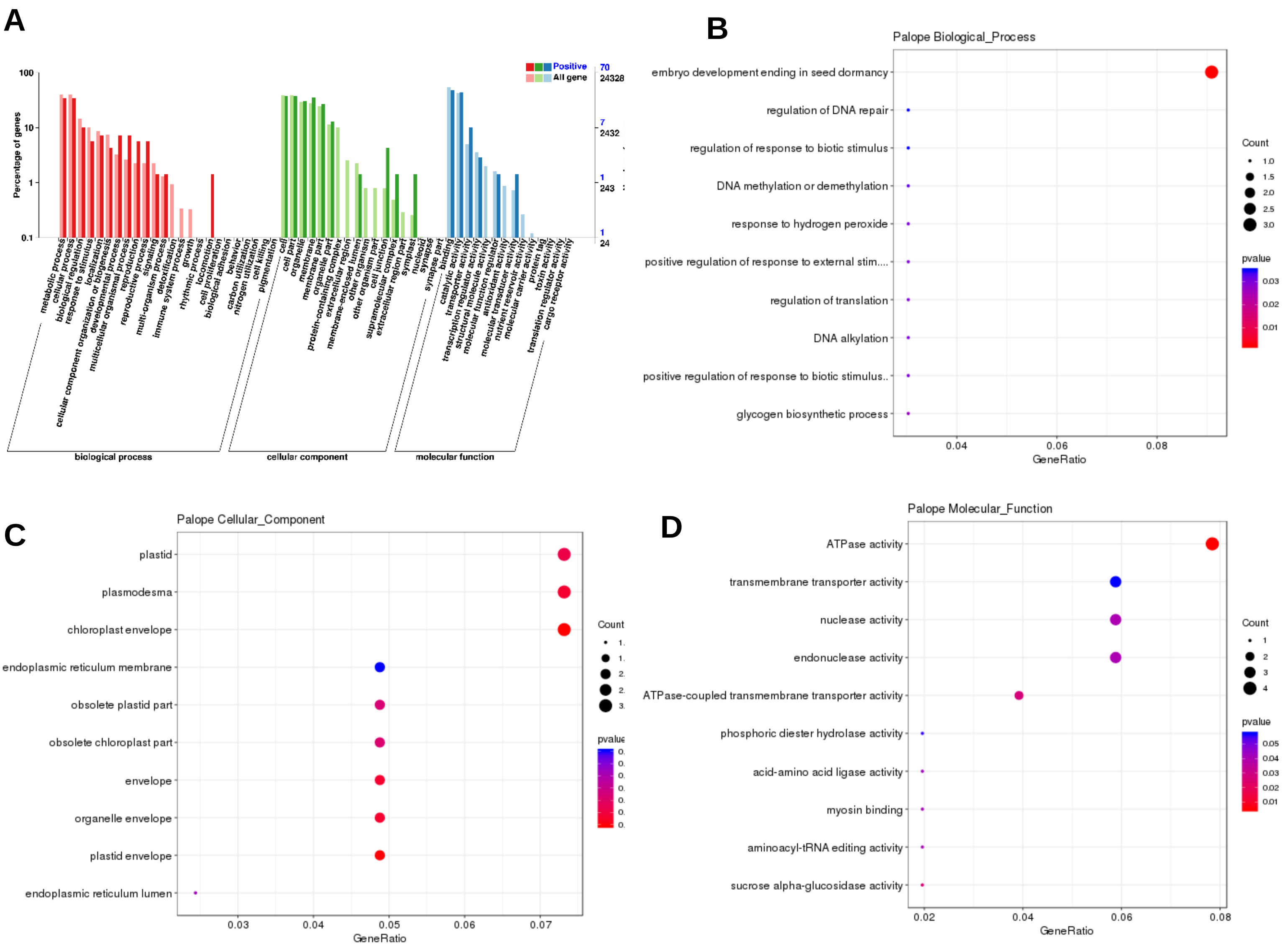

Figure S12 Enrichment analysis of the positive genes of *P. alopecuroides*. (A) GO enrichment analysis of endemic gene families. **(B)** The proportion of endemic genes to total endemic genes under biological process. **(C)** The proportion of endemic genes to total endemic genes under cellular component. **(D)** Pathways enriched in molecular function.
