# Supplementary material for: Chromosome-level reference genome assembly provides insights into the evolution of Pennisetum alopecuroides
Source: Front Plant Sci. 2023 Aug 23;14:1195479. doi: 10.3389/fpls.2023.1195479 (PMC10481962; doi:10.3389/fpls.2023.1195479)
Supplement: Supplementary file 13 [file DataSheet_13.pdf]

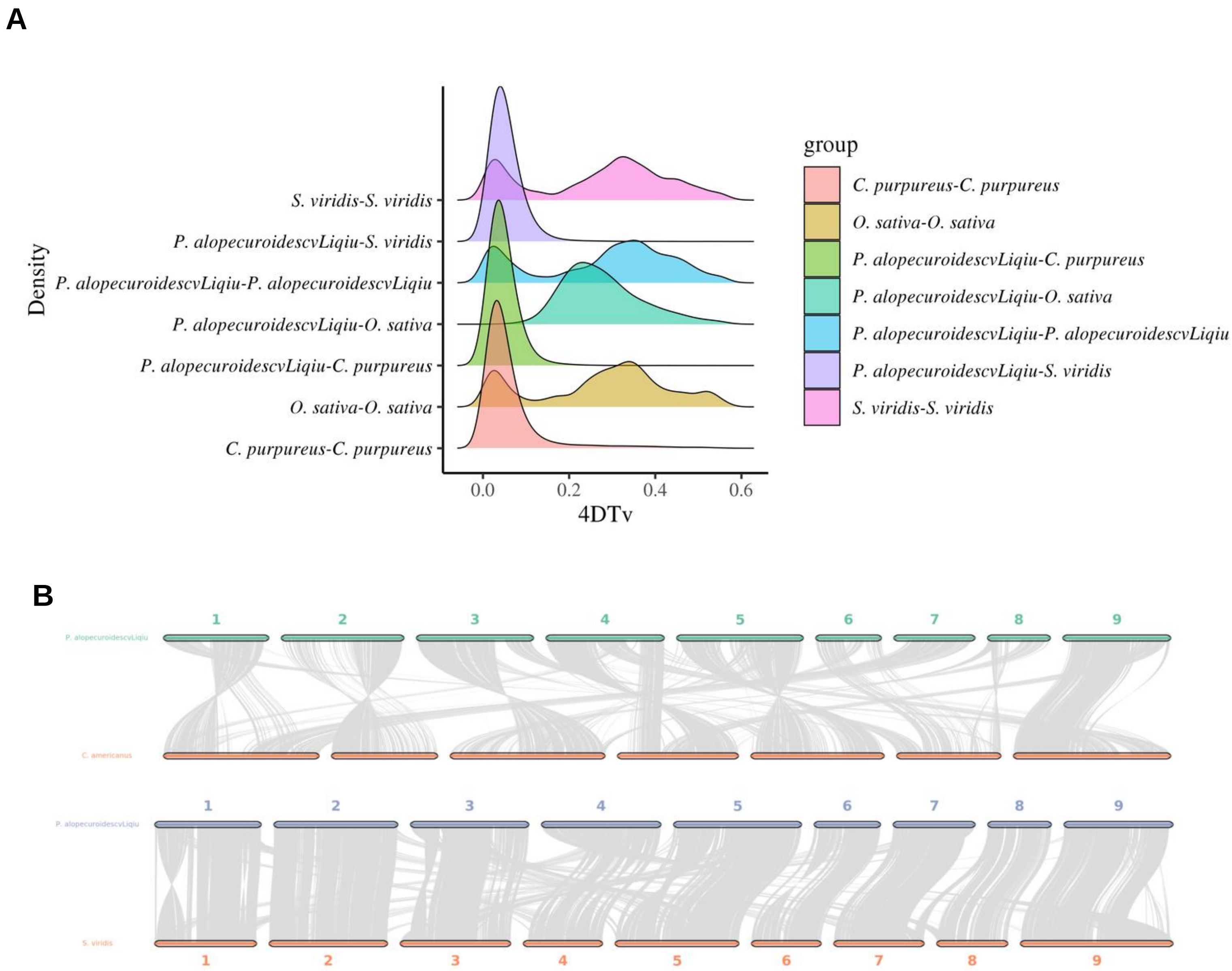

Figure S13 The analysis of collinearity *P. alopecuroides*. (A) 4DTv distribution map. The peaks of interspecies assemblages represent species divergence, and the peaks of intra-species assemblages represent genome-wide replication events of the species. **(B)** Gene collinearity relationship between *S. viridis* and *P. alopecuroides*, *C. americanus* and *P. alopecuroides*.
